# Aqueous extract of *Polygonum bistorta* modulates proteostasis by ROS-induced ER stress in human hepatoma cells

**DOI:** 10.1038/srep41437

**Published:** 2017-01-30

**Authors:** Yu-Huei Liu, Yui-Ping Weng, Hsuan-Yuan Lin, Sai-Wen Tang, Chao-Jung Chen, Chi-Jung Liang, Chung-Yu Ku, Jung-Yaw Lin

**Affiliations:** 1 Graduate Institute of Integrated Medicine, China Medical University, Taichung, 404, Taiwan; 2 Department of Medical Genetics and Medical Research, China Medical University Hospital, Taichung, 404, Taiwan; 3 Graduate Institute of Biological Science and Technology, Chung Hwa University of Medical Technology, Tainan, 717, Taiwan; 4 Department of Biological Science and Technology, Chung Hwa University of Medical Technology, Tainan, 717, Taiwan; 5 Department of Life Science, National Taiwan Normal University, Taipei, 116, Taiwan; 6 Institute of Biochemistry and Molecular Biology, College of Medicine, National Taiwan University, Taipei, 100, Taiwan

## Abstract

Hepatocellular carcinoma (HCC) remains the leading cause of cancer mortality with limited therapeutic targets. The endoplasmic reticulum (ER) plays a pivotal role in maintaining proteostasis in normal cells. However, alterations in proteostasis are often found in cancer cells, making it a potential target for therapy. *Polygonum bistorta* is used in traditional Chinese medicine owing to its anticancer activities, but the molecular and pharmacological mechanisms remain unclear. Using hepatoma cells as a model system, this study demonstrated that *P. bistorta* aqueous extract (PB) stimulated ER stress by increasing autophagosomes but by blocking degradation, followed by the accumulation of ubiquitinated proteins and cell apoptosis. In addition, an autophagy inhibitor did not enhance ubiquitinated protein accumulation whereas a reactive oxygen species (ROS) scavenger diminished both ubiquitinated protein accumulation and ligand-stimulated epidermal growth factor receptor (EGFR) expression, suggesting that ROS generation by PB may be upstream of PB-triggered cell death. Nevertheless, PB-exerted proteostasis impairment resulted in cytoskeletal changes, impairment of cell adhesion and motility, and inhibition of cell cycle progression. Oral administration of PB delayed tumour growth in a xenograft model without significant body weight loss. These findings indicate that PB may be a potential new alternative or complementary medicine for HCC.

Hepatocellular carcinoma (HCC) remains the leading cause of cancer mortality worldwide^1^,^2^,^3^,^4^,^5^. HCC patients usually present in advanced stages when surgical resection and/or chemical embolism are no longer feasible. Few chemotherapies and targeted therapies are capable of killing HCC. Therefore, new directions and new interventions are unmet urgent needs for HCC.

The endoplasmic reticulum (ER) plays an important role in maintaining proteostasis^6^. The accumulation of misfolded proteins in the ER initiates a protective unfolded protein response (UPR) in the cell in response to ER stress^7^. The major function of UPR is to modulate proteostasis through translational attenuation and upregulation of genes encoding ER chaperone proteins and secretory machinery to increase the protein-folding capacity of the ER^8^,^9^. However, persistent or intense stress will drive these unfolding proteins to translocate to the cytoplasm, where they are degraded through the ubiquitin-proteasome system (UPS). Once the UPS fails, death-associated protein kinase (DAPK), an upstream integrator of apoptosis and autophagy in response to ER stress, will be triggered^10^,^11^.

A significant drift has occurred in recent years towards the use of medicinal plants to manage/treat many debilitating diseases, including HCC^12^,^13^. PHY906, a 1800-year-old Chinese herbal formula, potentiates chemotherapy effects through improving multiple mechanisms, including the inflammatory state and tumour microenvironment^4^,^14^,^15^,^16^,^17^,^18^,^19^ and has been evaluated in clinical studies in colorectal cancer^20^,^21^, pancreatic cancer^22^,^23^, and is awaiting FDA approval for HCC^24^. The root and rhizome of *Polygonum bistorta* (pronounced *quansen*) has been a widely used Chinese medicine for more than 1000 years for treating exogenous heat, cough, dysentery, sepsis, haematemesis (blood heat bleeding), as well as inflammatory conditions such as carbuncle swelling and pain. Recent studies suggested that some organic solvent fractions of *P. bistorta* showed promise for the inhibition of xanthine oxidase activity^25^, therapeutic effects in snakebites^26^, ionophore-induced allergies^27^, retina ischemia/reperfusion injury^28^, and growth inhibition of various cancer cell lines including HCC lines *in vitro*^29^,^30^. Other new formulas including *P. bistorta* such as Zeng-Sheng-Ping (Antitumor B) for mutagen-induced lung cancer and oral cancer^31^,^32^, and Fei-Liu-Ping for lung cancer metastasis^33^,^34^, have claimed to be successful in the clinic for more than 20 years. However, the molecular and pharmacological mechanisms of *P. bistorta* require further investigation.

In the present study, we examined the efficacy of an aqueous extract of *P. bistorta* (PB) against hepatoma cells *in vitro*, as well as its antitumor effects on transplanted tumours in mice. We also demonstrate the anti-tumour mechanism of PB is mediated by reactive oxygen species (ROS), ER stress, autophagy, and proteasome degradation inhibition, which results in apoptosis.

## Results

### PB induces autophagy in Hep3B cells

**Figure 1:**
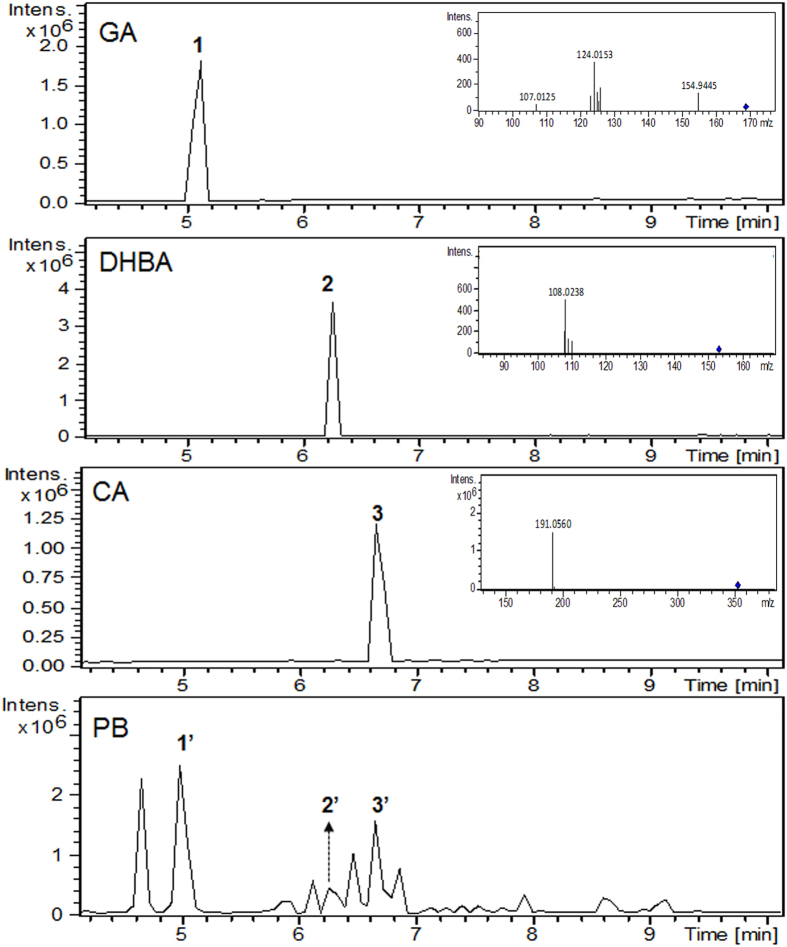
Chemical identification of polyphenols in PB. Chemical identification of polyphenols gallic acid (GA), 3,4-dihydroxybenzoic acid (DHBA), and chlorogenic acid (CA) in PB.

**Figure 2:**
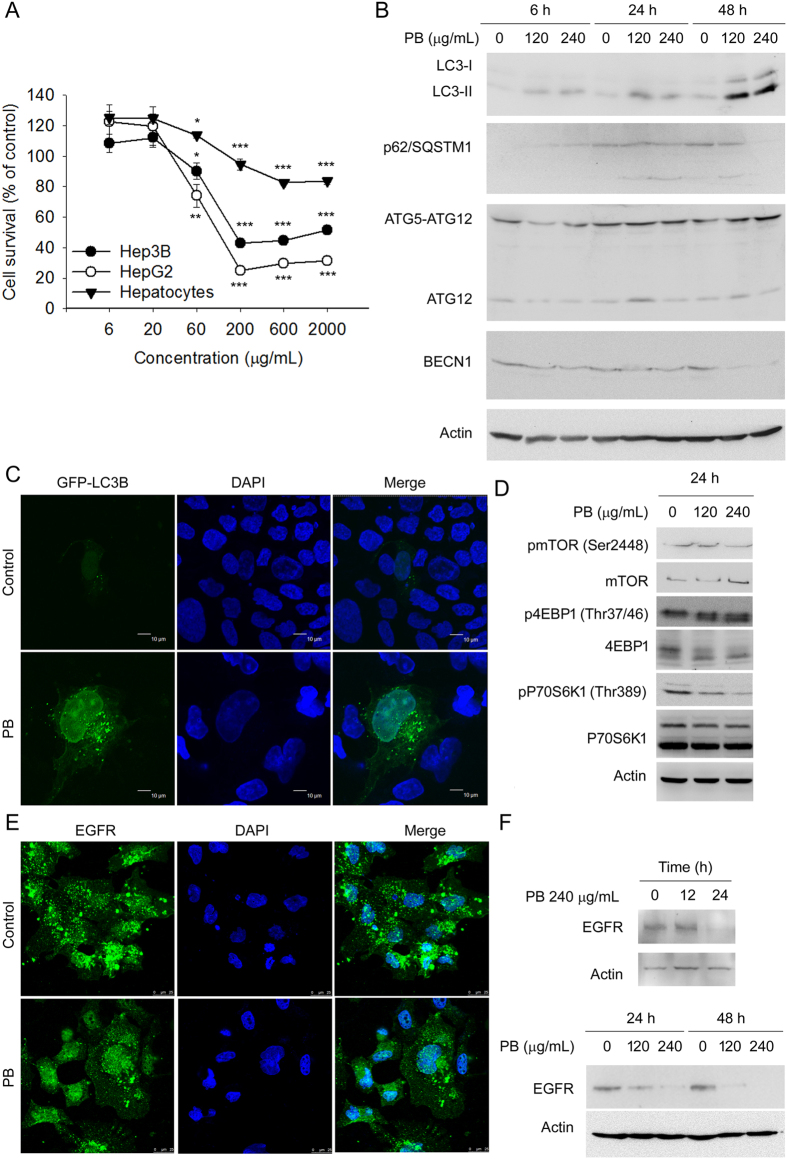
PB induces autophagy but blocks autophagosome trafficking and autophagosome-lysosome fusion in Hep3B cells. (**A**) Hep3B cells, HepG2 cells, and primary mouse hepatocytes were treated with increasing concentrations of PB (6–2000 μg/mL) for 24 h; viable cells were quantified using the MTT assay. Data are presented as mean ± SD from at least 3 independent experiments. Bars = SD. *P < 0.05, **P < 0.01, ***P < 0.001, control versus PB-treated cells. (**B**) Hep3B cells were treated for 6, 24 and 48 h with the indicated concentrations of PB. Levels of protein expression were analysed by Western blot using antibodies against LC3B, p62/SQSTM1, ATG12, BECN1 and Actin. Images were cropped from different blots run under the same experimental conditions. The original blots were attached as Supplementary Figure 3A. (**C**) Hep3B cells transfected with GFP-LC3B were treated with 240 μg/mL PB for 24 h and then observed under a confocal microscope. (**D**) Hep3B cells were treated for 24 h with the indicated concentrations of PB. Levels of protein expression were analysed by Western blot using antibodies against phosphorylated and total mTOR, 4EBP1, and p70S6K1 as well as actin. Images were cropped from different blots run under the same experimental conditions. The original blots were attached as Supplementary Figure 3B. (**E**) Hep3B cells were serum-starved overnight, and incubated with or without PB (240 μg/mL) for 6 h before stimulating with 20 ng/mL EGF for 20 min. EGFR protein levels were analysed by confocal microscope. (**F**) Hep3B cells were treated PB for 12, 24 or 48 h with indicated concentrations. Levels of protein expression were analysed by Western blot using antibodies against EGFR and Actin. Images were cropped from different blots run under the same experimental conditions in each panel. The original blots were attached as Supplementary Figure 3C.

Chemical profiling indicated that the quantities of gallic acid (GA), 3,4-dihydroxybenzoic acid (DHBA), and chlorogenic acid (CA), in PB were 2,091.4 ± 12.5 ppb, 162.6 ± 7.6 ppb, and 1,757.7 ± 170.7 ppb respectively, and were used as indicators for PB (Fig. 1). As shown in Fig. 2A, PB significantly decreased Hep3B and HepG2 cell survival in a dose-dependent manner. However, PB exhibited limited killing potency in mouse hepatocytes with identical treatment periods and concentrations. Administration of the polyphenols GA, DHBA, and CA at corresponding doses, either alone or in combination, did not show significant cytotoxic effects (data not shown), indicating that the complete PB extract is required to induce the cytotoxic effects.

The conversion from microtubule-associated protein 1 light chain 3b (MAP1LC3B/LC3B) (LC3B-I) to phosphatidylethanolamine covalent conjugated LC3B-II is a hallmark of autophagy^35^. To determine whether PB induced autophagy, the abundance of LC3B-I was monitored. As shown in Fig. 2B, PB increased the expression level of LC3B-II protein in a dose- and time-dependent manner in Hep3B cells. In addition, by monitoring autophagosome formation using a plasmid encoding GFP-LC3B fusion protein in Hep3B cells, GFP-LC3B puncta were observed in PB treated cells, indicating that PB treatment enhanced autophagosome accumulation in cells (Fig. 2C).

### PB facilitates autophagosome formation more rapidly than autolysosome formation in Hep3B cells

Autophagy is an intracellular process for the degradation of cytoplasmic contents by autolysosome fusion from isolated membrane (phagophore)-elongated autophagosomes and lysosomes^36^. Autophagosome accumulation in PB-treated cells reflects an imbalance between their rate of formation and degradation^37^, which could be explained by the promotion or impairment of autophagic flux. When examined using Western blot analyses, p62/SQSTM1, a marker to determine formation of autophagosome, was persistently increased in cells treated with PB at 6 h and 24 h, but not at the 48 h time point, implying that PB facilitated the accumulation of autophagosomes at least within 24 h of treatment. However, the expression level of an essential autophagic protein which mediates the maturation of autolysosome, beclin 1 (BECN1)^36^, was decreased by PB at all treatment concentrations and time points, indicating that autolysosome formation may be impaired by PB (Fig. 2B). Furthermore, when tracing the signalling for autophagosome initiation, we found that PB downregulated phosphorylated (p)-mechanistic target of rapamycin serine/threonine kinase (mTOR) and the surrogate measures of mTOR activity, p-p70S6K1 levels and expression of 4EBP1, indicating the involvement of mTOR inactivation by PB (Fig. 2D). These results demonstrated that PB downregulates mTOR activity to facilitate autophagosome formation more rapidly than autolysosome formation in Hep3B cells.

The impairment of autolysosome formation will cause cellular macromolecule proteolytic degradation dysfunction. Interference of autophagosome-lysosome fusion by sequestering two organelles in different localisations may be one cause for this impairment. Epidermal growth factor receptor (EGFR) is a typical receptor tyrosine kinase that is endocytosed and delivered from the cell surface to lysosomes for degradation after EGF binding-induced activation. To monitor lysosome localisation and activity, EGFR was chosen as an endogenous marker. Hep3B cells were first treated for 6 h with or without PB, and then EGF was added for 20 min. The localisation of EGFR was then observed. Interestingly, EGF-induced EGFR degradation was observed in the absence of PB treatment. However, EGFR appeared to translocate into the nucleus, implying that the dislocation of the lysosomes interfered with autolysosome formation (Fig. 2E). The rate of EGFR degradation was further monitored. Although PB did not alter the expression of EGFR at the 12 h treatment time point, it did downregulate EGFR at the 24 h and 48 h treatment time points, suggesting that PB-mediated lysosome nuclear localisation did not affect the degradation of endocytic cargo (Fig. 2F). Together, these results indicate that PB interferes with autophagosome fusion with lysosomes, or the formation of autolysosomes, but this interference has limited effects on the degradation of endocytic cargo.

### PB-induced autophagy and -obstructed UPS results in Hep3B cell apoptosis

**Figure 3:**
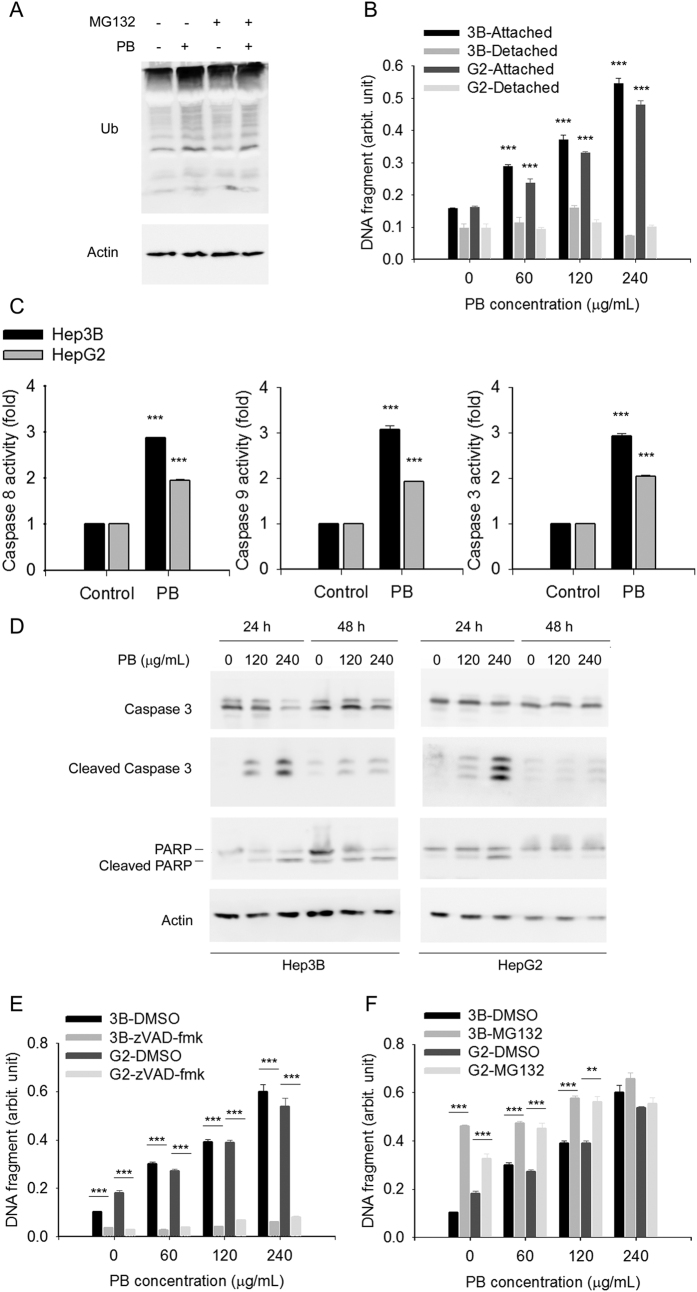
Simultaneous inhibition of autophagy and proteasome by PB triggers cell apoptosis. (**A**) Hep3B cells pre-treated with MG132 were treated for 6 h with the indicated concentrations of PB. Levels of protein expression were analysed by Western blot using antibodies against Ub and Actin. Images were cropped from different blots run under the same experimental conditions. The original blots were attached as Supplementary Figure 4A. (**B**) Hep3B and HepG2 cells treated for 24 h with indicated concentrations of PB. Fragment DNA in attached cells and medium was detected using ELISA kit. Data are presented as mean ± SD for 3 independent experiments. ***P < 0.001, control versus PB-treated cells. (**C**) Hep3B and HepG2 cells treated for 24 h with indicated concentrations of PB. Caspase activity of caspase 9, caspase 8, and caspase 3 were measured using specific chromo-substrates. Data are presented as mean ± SD for 3 independent experiments. ***P < 0.001, control versus PB-treated cells. (**D**) Hep3B and HepG2 cells were treated for 24 h with the indicated concentrations of PB. Levels of protein expression were analysed by Western blot using antibodies against intact or cleaved forms of caspase 3 and PARP as well as Actin. Images were cropped from different blots run under the same experimental conditions. The original blots were attached as Supplementary Figure 4B. (**E**) Hep3B and HepG2 cells pretreated with zVAD-fmk were treated for 24 h with indicated concentrations of PB. Fragment DNA in attached cells and medium was detected using ELISA kit. Data are presented as mean ± SD for 3 independent experiments. ***P < 0.001, control versus PB-treated cells. (**F**) Hep3B and HepG2 cells pretreated with MG132 were treated for 24 h with indicated concentrations of PB. Fragment DNA in attached cells and medium was detected using ELISA kit. Data are presented as mean ± SD for 3 independent experiments. **P < 0.01, ***P < 0.001, control versus PB-treated cells.

UPS is another cellular protein degradation system that is independent of lysosomal degradation. UPS and autophagy are interdependent^38^,^39^. Inhibition of autophagy will result in ubiquitinated protein aggregation^39^. Indeed, ubiquitinated protein levels were increased by PB, indicating that PB inhibited proteasome activity (Fig. 3A). In addition, the proteasome formation inhibitor MG132 did not significantly aggravate PB-augmented accumulation of ubiquitinated proteins (Fig. 3A), suggesting that PB itself saturates the ability of MG132 to inhibit protein degradation. These results indicated that PB also obstructed protein degradation through lysosome-independent UPS.

We next determined whether PB-induced autophagy and -obstructed UPS were associated with apoptosis. DNA fragmentation assays indicated that PB increased cellular apoptosis of attached cells in a dose-dependent manner, but only exerted limited effects in detached cells, suggesting that little anoikis occurred (Fig. 3B). In addition, PB effectively induced activities of caspase 8, caspase 9, and caspase 3 (Fig. 3C), indicating both extrinsic and intrinsic apoptotic pathways were activated. Western blotting further demonstrated increased expression levels of cleaved poly (ADP-ribose) polymerase 1(PARP1) and caspase 3 (Fig. 3D). Pretreatment of cells with a pan-caspase inhibitor, zVAD-fmk, almost completely blocked PB-induced DNA fragmentation (Fig. 3E). In contrast, pretreatment of cells with MG132 enhanced 60 and 120 μg/mL, but not 240 μg/mL, PB-induced DNA fragmentation (Fig. 3F). These results indicate that PB-induced autophagy and -obstructed UPS may subsequently trigger caspase-dependent apoptosis in Hep3B cells.

### PB-triggered apoptosis occurred in an ER stress-related manner in Hep3B cells

**Figure 4:**
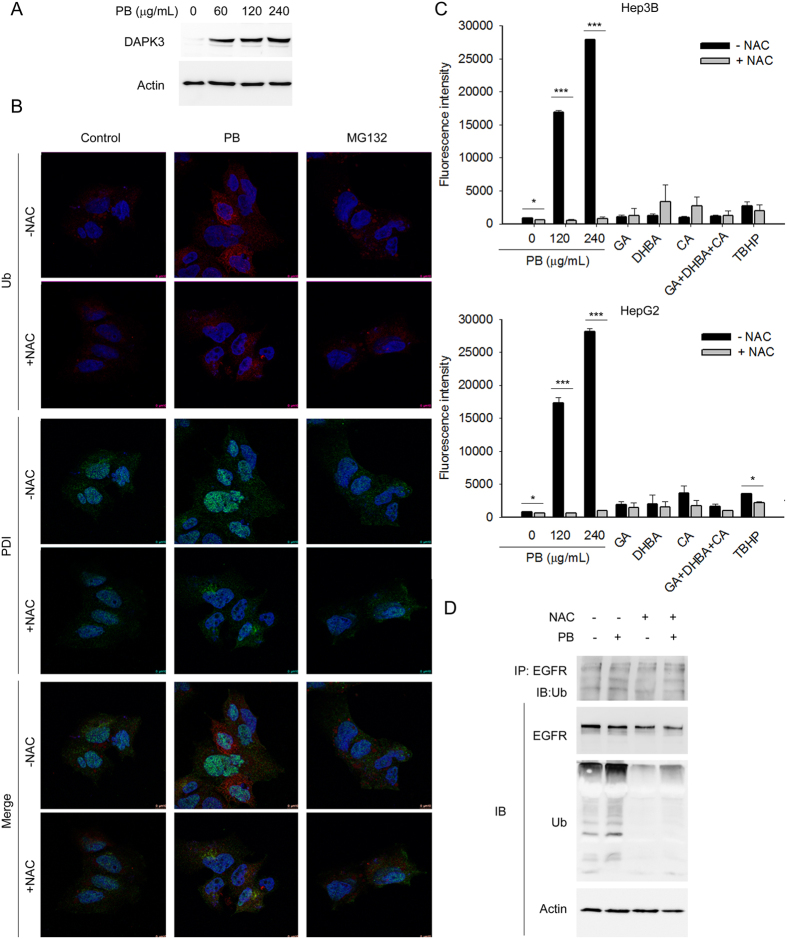
PB triggered cell apoptosis involves elevation of reactive oxygen species (ROS) and accumulation of ER stress. (**A**) Hep3B cells were treated for 6 h with the indicated concentrations of PB. Levels of protein expression were analysed by Western blot using antibodies against DAPK3 and Actin. Images were cropped from different blots run under the same experimental conditions. The original blots were attached as Supplementary Figure 5A. (**B**) Localisation of the ubiquitinated protein aggregates and ER stress and ROS-related protein PDI was imaged using a confocal microscope (Leica SP8). (**C**) Hep3B or HepG2 cells pretreated with or without 1 h N-acetyl-L-cysteine (NAC, 5 mM) were treated for 5 h with PB, or polyphenols gallic acid (GA), 3,4-dihydroxybenzoic acid (DHBA), and chlorogenic acid (CA), alone or in combination. Total reactive oxygen species (ROS) generation was quantified using DCFDA. Tert-butyl hydrogen peroxide (TBHP, 20 μM) was used as a positive control. Data are presented from three independent experiments. *P < 0.05, ***P < 0.001, NAC treated versus NAC untreated reactions. (**D**) Hep3B cells pre-treated with NAC were treated for 6 h with the indicated concentrations of PB. Levels of protein expression were analysed by Western blot using antibodies against Ub and Actin. Images were cropped from different blots run under the same experimental conditions. The original blots were attached as Supplementary Figure 5B.

General agreement suggests that one purpose for the upregulation of autophagy is to remove misfolded protein aggregates to ameliorate ER stress^40^,^41^,^42^. Once this fails, cells undergo apoptosis to minimize systemic damage. Death-associated protein kinase 3 (DAPK3) is an upstream integrator of apoptosis and autophagy in response to ER stress^11^. Indeed, DAPK3 was dramatically upregulated upon PB treatment, implying the involvement of ER stress in PB cytotoxic effects (Fig. 4A). To investigate whether ER stress was involved in PB-induced proteasome inhibition, cells treated with or without PB were stained with ubiquitin (Ub) and a dual functional protein disulphide isomerase (PDI), which has chaperone activity in the ER but redox properties outside the ER, and compared to those treated with MG132. As expected, confocal microscopy demonstrated different Ub distribution patterns in cells: a light vacuole pattern in untreated cells compared to a dense reticular pattern in PB treated cells. In addition, PDI showed a reticular pattern in untreated cells but a nuclear localisation pattern in PB treated cells. No similar observations were obtained in the MG132 group (Fig. 4B). ROS are frequently induced by herb-induced ER stress to synergistically trigger protein degradation inhibitor-mediated autophagy and apoptosis^43^. Induction of autophagy and obstruction of UPS may also induce intracellular ROS production. Therefore, the status of PB-induced ROS was measured. As expected, PB treatment induced high levels of ROS in Hep3B and HepG2 cells. Pretreatment of cells with a free radical scavenger NAC significantly reduced PB-induced ROS production (Fig. 4C) and PB-induced ubiquitinated protein accumulation (Fig. 4D), indicating NAC reduced the ER stress induced by PB. In addition, pretreatment with NAC downregulated endogenous EGFR and PB-induced ubiquitinated EGFR (Fig. 4D). These results suggested that PB deregulates proteasome formation and induces cell death in part via ROS-induced ER stress.

### PB inhibits cell cycle progression, cell-cell adhesion, and cell migration through the disruption of actin cytoskeleton organization

**Figure 5:**
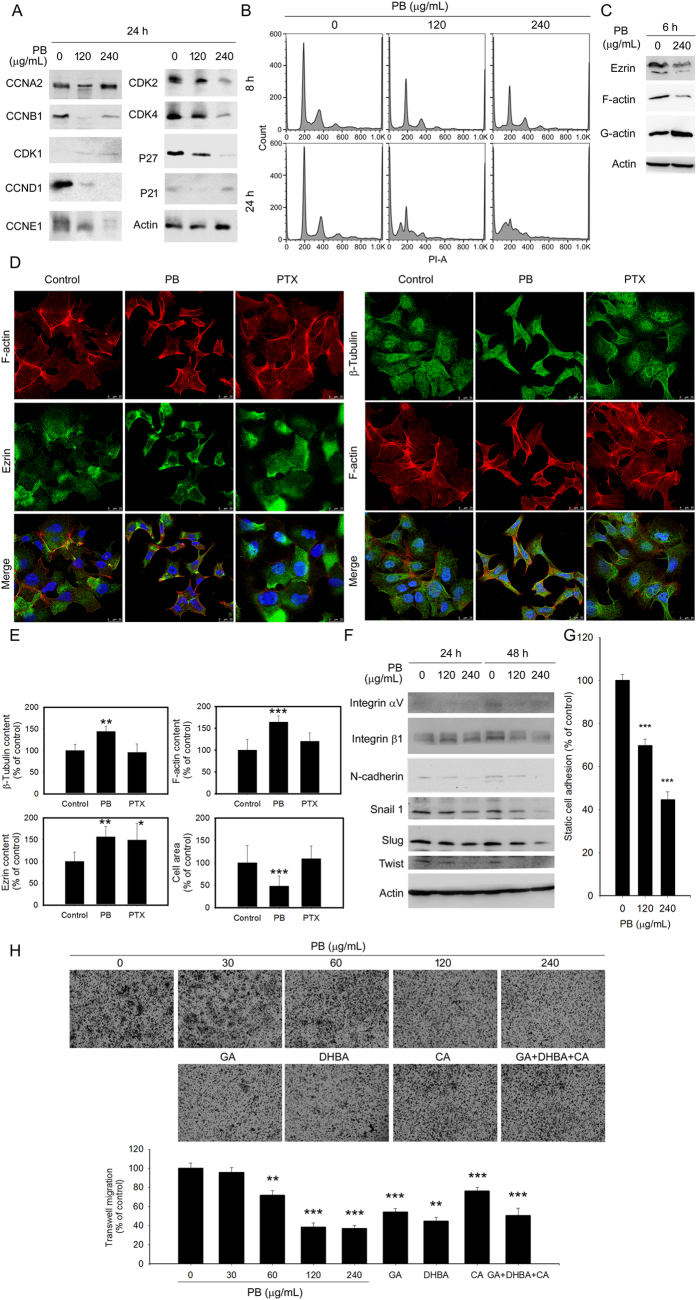
PB downregulates several proteins related to cell cycle progression, morphology, cell-cell adhesion and cell migration. Hep3B cells were treated for 24 h (**A**), 6 h (**C**), and 24 h and 48 h (**F**) with the indicated concentrations of PB. Levels of protein expression were analysed by Western blot using indicated antibodies. Images were cropped from different blots run under the same experimental conditions in each panel. The original blots were attached as Supplementary Figure 6. (**B**) Hep3B cells were treated for 8 or 24 h with 120 or 240 μg/mL PB. Cell cycle distribution was evaluated using propidium iodide (PI) staining. (**D**,**E**) Hep3B cells were treated for 6 h with PB (120 μg/mL), paclitaxel (PTX) (2.0 μM). Localisation of β-tubulin, F-actin and ezrin was imaged using a confocal microscope (Leica SP8). Differential expression of indicated molecules was compared. *P < 0.05; **P < 0.01, ***P < 0.001, control versus PB- or drug-treated cells. (**G**) Hep3B cells were treated for 1 h with indicated concentration of PB and adhered to the bottom of the plates. Adhesive cells were photographed and counted. (**H**) Hep3B cells were treated for 24 h with PB (60–240 μg/mL), the corresponding concentration of polyphenols GA, DHBA, and CA, along or in combination, or water control. Migration analysis was performed using a Boyden chamber. Migrated cells were measured as a percentage of cells that migrated to the lower surface of the chamber. Data are presented from three independent experiments. **P < 0.01; ***P < 0.001, control versus PB- or drug-treated cells.

To examine the potential cancer killing potential of PB, two main mechanisms of cancer progression, cell cycle progression and cytoskeleton organization, were examined. Western blotting revealed that PB decreased cyclin and cyclin dependent kinase (CDK) levels in Hep3B and HepG2 cells (Fig. 5A). Flow cytometry revealed that PB treatment significantly increased apoptotic sub-G1 phase content, indicating an increase of DNA fragments after PB treatment (Figs 3C and 5B). These results indicate that PB deregulates the expression of cyclins and CDKs to perturb cell cycle progression, leading to hepatoma cancer cell apoptosis.

Actin and microtubules provide a dynamic cellular framework to maintain and orchestrate cellular processes including motility. Indeed, downregulation of filament actin (F-actin) and its cell membrane-associated scaffold protein, Ezrin, was observed concomitantly after PB treatment (Fig. 5C). In addition, confocal microscopy analysis showed that PB treatment induced the alteration of F-actin and β-tubulin cytoskeleton organization in Hep3B cells (Fig. 5D and E). Furthermore, integrin αVβ1, which mediates actin cytoskeletal remodelling in response to force transmission orientation and dynamic stiffening or cyclic stretching of the extracellular matrix^44^, as well as N-cadherin and the transcription factors twist, slug, and snail, were all down-regulated in response to PB (Fig. 5F).

Nevertheless, functional analyses revealed that PB significantly restricted cell adhesion and migration in a dose-dependent manner (Fig. 5G and H). Wound closure following PB or control treatment were recorded using time-lapse microscopy, showing that PB significantly inhibited wound closure from 6 h after treatment (Supplementary Fig. S1A). The velocity and migrated distance of PB-treated cells were significantly reduced when compared with control-treated cells and even microtubule-stabilizing agent PTX-treated cells. Interestingly, the polyphenols GA, DHBA, and CA, alone or in combination, were involved in PB-restricted Hep3B cell migration (Supplementary Fig. S1B). These results show that PB restricts cancer cell adhesion and motility through PB-disrupted proteostasis. In addition, the polyphenols GA, DHBA, and CA also contribute to the inhibitory effects of PB on motility.

### PB inhibits Hep3B and HepG2 cell growth in NOD-SCID xenograft mice

**Figure 6:**
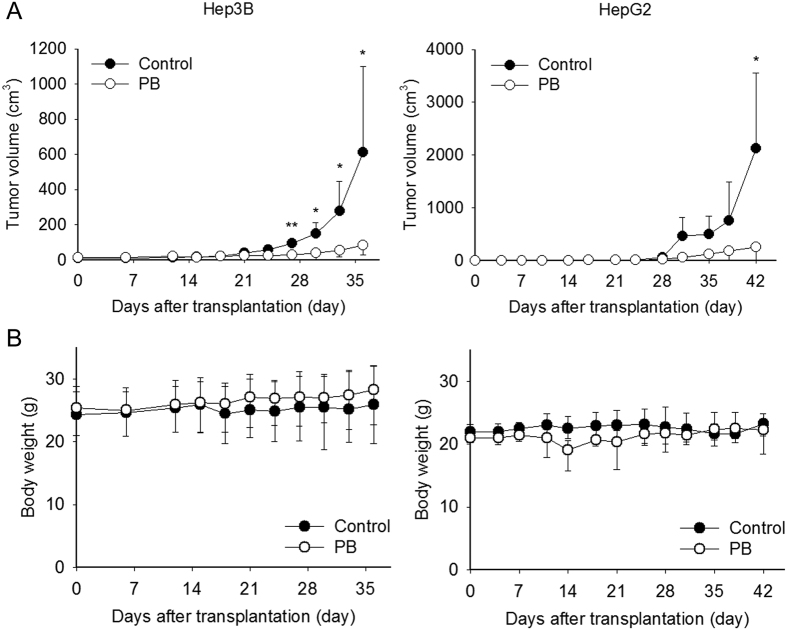
PB inhibits Hep3B and HepG2 xenograft growth in NOD-SCID mice without apparent toxicity. (**A**) Hep3B and HepG2 bearing mice were orally administrated either water or PB (5.8 mg lyophilised PB/100 μL of water/day) for 4 or 5 weeks. Tumour growth following PB treatment was significantly delayed in both Hep3B and HepG2 xenograft mice. (**B**) No difference in body weight was observed between Hep3B or HepG2 xenograft mice treated with (open circle) and without (closed circle) PB. Data are presented from at least four mice per group. *P < 0.05.

To translate the anti-HCC effects of PB *in vivo*, Hep3B and HepG2 xenograft mouse models were utilised. At 7 days post-cancer cell injection, mice were randomly assigned to vehicle or PB groups. All treatments were orally administered five times per week for 25 days. As demonstrated in Fig. 6A, PB administration significantly reduced tumour volume when compared with vehicle group in both Hep3B xenograft mice at day 35 (P = 0.045) or HepG2 xenograft mice at day 42 (P = 0.043). No significant weight loss was observed (Fig. 6B). These results indicate that PB potently attenuates tumour growth in Hep3B- and HepG2-bearing mice cells *in vivo*, without exerting severe toxic effects.

### Transcriptome regulation by PB in Hep3B and HepG2 cells

To further examine the effect of PB in Hep3B and HepG2 cells, PB-induced changes in gene expression were determined by microarray analysis using a whole genome Human OneArray^®^. Genes significantly up- or down-regulated (P < 0.05) by PB are listed in Supplementary Table S1. The 50 genes that were most highly upregulated and most heavily downregulated by PB treatment were chosen to construct a heat map (Supplementary Fig. 2A). The enriched top 5 association networks are listed in Table 1, and the top 1 network is shown in Supplementary Fig. 2B. CDK1 was shown as a key regulator in network 1. In addition, molecular analysis revealed that 11 upregulated and 7 downregulated molecules could be applied as biomarkers for diagnosis, therapeutic efficacy, disease progression, safety, prognosis, and response to therapy. Drug analysis revealed that PB has multiple targets, including TUBB3, AGER, DCK, and TOP2A, implying the potential of PB as an alternative medicine. Nevertheless, drug analysis further indicated the potential for co-treatment with PB and a SERPINE1 inhibitor such as drotrecogin alfa to enhance anti-cancer effects (Supplementary Table S2). The networks generated by Ingenuity pathway analysis software further supported that PB suppressed cancer through the inhibition of cellular growth and proliferation, inhibition of cellular development, promotion of cell death, disruption of the cell cycle, and blockage of cellular movement. In addition, it provided potential clinical applications that may merit further translational study.

## Discussion

In the present study, we report that PB elicited ER stress, which inhibited autophagosome and proteasome activity, resulting in apoptosis induction in Hep3B cells. In addition, cell adhesion and motility were restricted through the inhibition of integrins and cadherins, as well as the modulation of actin cytoskeleton organization. Furthermore, PB inhibited tumour growth without affecting body weight in xenografts. These results suggest inhibition of the autophagy and ubiquitin-proteosome systems rendered cells vulnerable to PB-initiated ER stress-triggered apoptosis. Our results agree with those of the previous reports for the chemicals present in this plant and further elucidate the cancer inhibitory effects of this plant extract^29^,^30^.

The relationship between autophagy and apoptosis is intricate. Accumulation of autophagy could induce the bulk degradation process, leading to apoptosis, whereas autophagy will be negatively regulated by the initiation of apoptosis^45^,^46^,^47^,^48^. We found that PB treatment for 6 h initiated the formation of autophagosomes and increased the expression of LC3B-II without inducing apoptosis. However, PB activated apoptotic pathways after 24 h treatment. Our results showed that PB-inhibited autophagy enhanced apoptosis and the anti-tumour effects of PB *in vitro* and *in vivo*.

The alternation of growth factors, cytoskeletons, and adhesion molecules during proteasome formation is considered as one strategy to change cell mobility and adhesion^49^,^50^. Indeed, more than 50% of clinical Western medicines originate from natural products, many of which have the ability to modulate cancer cells^51^. In addition, ROS has been shown to medicate chemopreventive agent-induced cancer cell death^52^. In this study, we found marked downregulation of EGFR, F-actin, and ezrin and reorganization of F-actin network, as well as downregulation of adhesion molecules and their upstream transcription factors in response to PB treatment. Moreover, the role of ROS in the induction of ER stress, followed by a dysregulation of proteostasis and subsequent apoptosis is expected^53^. Elevation of ROS leads to protein damage and degradation, but cancer cells may counteract stress through increasing their antioxidant defences to eliminate ROS^54^ or restore redox balance^55^, implying that PB may lower cancer cell antioxidant defences and thereby increasing the antitumor growth rate. These findings demonstrate that the anti-proliferative and anti-migratory activities of PB are at least in part due to ROS elevation. Future work should concern the development of an ideal PB-based anti-cancer formula based on the rules of the monarch/minister/assistant/guide in traditional Chinese medicine theory.

The therapeutic dose information generated in mice in this study could serve as a reference for future clinical studies by conversion to equivalent human intake information using the body surface area normalization method^56^. According to the conversion method, 276 mg/kg/day of PB in mice (~21 g body weight on average) would be ~1.4 g/day in human adult with 60 kg [276 mg/kg × 60 kg × Km factor for mice (3)/Km factor for humans (37)]^57^. This could be supplied by the consumption of herb extracts via dietary supplements.

In summary, we have demonstrated that PB induces cell death, inhibits cell adhesion, and restricts cell motility through a mechanism likely to involve activation of proteasome degradation and inhibition of protein synthesis in Hep3B cells. The present study provides new insights into the application of PB for HCC treatment.

## Methods

### Preparation and liquid chromatography–mass spectrometry/mass spectrometry (LC-MS/MS) analysis of PB

Aqueous extract of *P. bistorta* was obtained from Sun Ten Pharmaceutical Company (Taipei, Taiwan). First, 100 g of *P. bistorta* was boiled with 1.5 L of H_2_O at 100 °C for 30 min, then concentrated to 100 mL under reduced pressure, designed as *P. bistorta* soup. A clear supernatant was obtained by centrifugation at 12,000 rpm for 20 min and designed as PB. PB was estimated to contain a total of 58 mg of residues per mL by concentration in vacuo.

Gallic acid (GA, G7384), 3,4-dihydroxybenzoic acid (DHBA, 37580), chlorogenic acid (CA, C3878), and all other chemicals used were of analytical grade and obtained from Sigma-Aldrich. Retention time and MS/MS ion spectra of the standards were used to confirm polyphenol presence in PB extracts. The quantitation of the polyphenols in PB was performed using a standard addition method with LC-MS/MS analysis by monitoring their precursor ions. Briefly, a high-performance liquid chromatographic system (Ultimate 3000 LC; Dionex, Germany) coupled with a hybrid Q-TOF mass spectrometer (maXis impact; Bruker, Taiwan Co. Ltd) was applied, with chromatographic separation using an Atlantis T3 analytical column (C18, 5 μm, 2.1 × 150 mm; Waters, Millford, MA, USA). Mobile phase A consisted of 5% acetonitrile/0.1% formic acid, and mobile phase B consisted of acetonitrile/0.1% formic acid. A linear LC gradient was used from 5% (v/v) B to 99% B at a flow rate of 0.25 mL/min for 15 min. Between injection, a program consisting of a 9 min 99% phase B followed by a 40 min 1% phase B (v/v) was used to re-equilibrate the column. The ESI source was operated in the negative ion mode. Nitrogen was used for nebulizing (50 psi) and drying (8 L/min, 350 °C), and helium was used for collision. MS and MS/MS data were processed and extracted using DataAnalysis software 4.1 (Bruker).

### Cell culture

All culture media were purchased from Thermo Fisher Scientific. The human hepatoma cancer cell lines Hep3B and HepG2, or primary mouse hepatocytes isolated from NOS-SCID mice were maintained respectively in Roswell Park Memorial Institute (RPMI) 1640 (31800-022), RPMI 1640, or Dulbecco’s modified Eagle medium (DMEM) (12100046) with 10% foetal bovine serum (16000044), 50 U/mL penicillin and 50 μg/mL streptomycin (15070063), and 2 mM L-glutamine (25030081) at 37 °C in a humidified atmosphere of 5% CO_2_.

### Cell viability and imaging

5 × 10^3^ Hep3B or HepG2 cells were seeded into each well of 96-well plates for 24 h. Then, the culture medium was replaced by the medium containing PB (0–2000 μg/mL), and the volume of each well is 100 μL. Following a further 24 h of culture, viable cells were estimated by measuring the conversion of 3-(4,5-dimethylthiazol-2-yl)-2,5-diphenyltetrazolium bromide (MTT) (M2128, Sigma-Aldrich) to formazan crystals. Cell images were photographed using a live cell movie analyser (NanoEntek, Seoul, Korea).

### Western blot analysis

Cells were lysed with lysis buffer (10 mM Tris, pH 7.5; 150 mM NaCl; 5 mM EDTA, pH 8.0; 0.1% sodium dodecyl sulphate (SDS); 1% deoxycholate; and 1% NP-40) supplemented with protease inhibitor cocktail (Roche). Cell lysates were subjected to SDS-polyacrylamide gel electrophoresis and then transferred to polyvinylidene fluoride membranes. After blocking with 5% skim milk, the membranes were incubated with primary antibodies and subsequently with appropriate peroxidase-conjugated secondary antibodies. Primary antibodies specific to p-mTOR, mTOR, p-4EBP1, p-p70S6K1 (#9862), 4EBP1 (#9452), p70S6K1 (#9202), caspase 3, cleaved caspase 3, poly (ADP-ribose) polymerase (PARP), cleaved PARP (#9915), cyclin B1 (CCNB1), CCND1, CCNE1 (#9869), ezrin (#3145), N-cadherin (#9961), integrin αV and integrin β1(#4749) were purchased from Cell Signaling Technology. Antibodies specific to EGFR (sc-03) and Ub (sc-8017) were purchased from Santa Cruz. Antibodies specific to Atg12 (GTX124181), p62/SQSTM1 (GTX100685), DAPK3 (GTX102404), CCNA2 (GTX103042), CDK1 (GTX110545), CDK2 (GTX101226), CDK4 (GTX102993), p27 (GTX100446), p21 (GTX100444), Snail1 (GTX100754), Slug (GTX121924), and Twist (GTX127310) were obtained from GeneTex, CA, USA. Antibodies specific to LC3 were purchased from Novus Biologicals, CO, USA (NB600-1384). Antibodies specific to actin were obtained from Millipore, CA, USA (MAB1501). Peroxidase-conjugated secondary antibodies were obtained from Jackson ImmunoResearch, PA, USA. Blots were developed using an enhanced chemiluminescence system (Thermo Fisher Scientific).

### Immunofluorescence cell staining

For detecting LC3 pattern, Hep3B cells were transfected with pEGFP-LC3 (#24920, Addgene) for 24 h followed by treatment with PB (240 μg/mL) or water control for a further 24 h. For detecting EGFR distribution, cells were seeded onto four-chamber slides, treated accordingly, and fixed with 4% paraformaldehyde in PBS (phosphate-buffered saline, 137 mM NaCl, 2.7 mM KCl, 10 mM Na_2_HPO_4_, 2 mM KH_2_PO_4_, pH 7.4), permeabilised with 0.1% Triton X-100 (X100, Sigma-Aldrich) in PBS, rinsed with PBS, and blocked with 5% bovine serum albumin (A2153, Sigma-Aldrich) in PBS and stained with primary antibodies for EGFR (sc-03, Santa Cruz), ezrin (#3145, Cell Signaling Technology), β-tubulin (sc-9104, Santa Cruz), or Ub (sc-8017, Santa Cruz), followed by Alexa-488 or Alexa-594-conjugated secondary antibody (ThermoFisher Scientific). Filament actin (F-actin) was stained with Alexa-594 conjugated phalloidin (A12381, ThermoFisher Scientific), and nuclei were stained using 4′,6-diamidino-2-phenylindole (DAPI) (D9542, Sigma-Aldrich). N-acetyl-L-cysteine (NAC) (A7250, Sigma-Aldrich) and Paclitaxel (PTX; 2.0 μM) were used accordingly. Fluorescence imaging was taken using a confocal laser scanning microscope (Leica TCS SP8, Leica microsystems, Wetzlar, Germany). Fluorescence intensities indicating an abundance of β-tubulin, F-actin, and ezrin were quantified using ImageJ software (National Institutes of Health, USA).

### Detection of cell apoptosis

Cell apoptosis was quantitatively and qualitatively analysed by the following approaches: (i) Measure DNA fragmentation and histone release from nucleus using the Cell Death Detection ELISA PLUS kit (11774425001, Roche Diagnostics Ltd., Taipei, Taiwan) according to the manufacturer’s instructions. (ii) Caspase activity measurement of caspase 9, caspase 8, and caspase 3 using a Colorimetric Assay Kit according to the manufacturer’s instructions (BioVision) with LEHD-*p*NA (K119), IETD-*p*-nitroaniline (*p*NA) (K113), and DEVD-*p*NA (K106) as substrates, respectively. Fold-increase in caspase activity was determined by colorimetric signal normalised to cell viability followed by comparison of the results of treated samples with that of controls.

### Cell cycle analysis

5 × 10^6^ Hep3B cells were treated with PB for 8, 24, or 48 h, and then pelleted, and fixed overnight in ice-cold 70% ethanol in PBS. Cells were then washed twice with PBS and resuspended in 0.5 mL staining buffer consisted of 50 μg/mL propidium iodide (PI) (P4170, Sigma-Aldrich) and 10 μg/mL RNase A (R6513, Sigma-Aldrich) in PBS for 1 h incubation at room temperature in the dark, and followed by analysing using a FACSCaliber™ platform (Becton Dickinson Immunocytometry Systems, CA, USA). Data analysis was performed using FlowJo software (FlowJo, LLC, OR, USA).

### Cell adhesion analysis

5 × 10^5^ Hep3B cells were treated with indicated concentration of PB and seeded on 6-well plate for 1 h to allow the cells to adhere to the bottom of the plates, and the wells were washed with PBS and the cells were fixed in 4% paraformaldehyde. Adhesive cells were photographed and counted.

### Transwell cell migration assay

24 h growth factor-reduced Hep3B cells were suspended in RPMI-1640 with 1% serum, and 1 × 10^5^ cells were seeded into transwell Boyden chambers (8 μm pore, Millipore). Medium with 10% serum with PB (60–240 μg/mL), or a corresponding concentration of GA, DHBA, CA, or water control, was added into the lower chamber, and cells were cultured for 24 hrs. After fixation with 10% formaldehyde, cells were stained with 0.5% crystal violet (C0775, Sigma-Aldrich). Migrated cells on the underside of the membrane were photographed using the live cell movie analyser and evaluated using the Cell Counter plugin for ImageJ software.

### Measurement of reactive oxygen species (ROS)

Cellular ROS was measured in Hep3B or HepG2 cells treated with PB (120 and 240 μg/mL), corresponding polyphenols, or water control for 5 h, with or without 1-h pretreatment with 5 mM NAC, by using a 2′-7′-dichlorofluorescin diacetate (DCFDA)-Cellular Reactive Oxygen Species Detection Assay Kit (ab113851, Abcam) according to the manufacturer’s protocol. Cells were analysed on a fluorescent plate reader (Berthold Technologies, TN, USA), and mean ± standard deviation was plotted for three replicates from each condition. Tert-butyl hydrogen peroxide (TBHP), which mimics ROS activity to oxidize DCFDA to fluorescent DCF, was used as positive control.

### Mouse xenograft model

Five-week-old, female, non-obese diabetic/severe combined immunodeficiency (NOD-SCID) mice were obtained from Bio-LASCO Taiwan Co. Ltd (Taipei, Taiwan). All animal experiments were carried out according to regulations approved by the Laboratory Animal Center, National Taiwan University 20120013. For tumour growth experiments, 5 × 10^6^ Hep3B or HepG2 cells were inoculated subcutaneously into the flanks of mice. Seven days after inoculation, mice were randomized into two groups and orally administered PB (232 mg/kg, n = 5 for Hep3B, n = 4 for HepG2) or water (n = 6 for Hep3B, n = 4 for HepG2), five times per week for a further 28 days (Hep3B cells) or 35 days (HepG2 cells). Tumour size was measured by calipers every 3 days, and tumour volume was determined using the following formula: width^2^ × length/2.

### Statistical analysis

Statistical significance of differences between treatments was determined by two tailed Student’s *t* test. P < 0.05 was considered statistically significant.

## Additional Information

**How to cite this article:** Liu, Y.-H. *et al*. Aqueous extract of *Polygonum bistorta* modulates proteostasis by ROS-induced ER stress in human hepatoma cells. *Sci. Rep.* **7**, 41437; doi: 10.1038/srep41437 (2017).

**Publisher's note:** Springer Nature remains neutral with regard to jurisdictional claims in published maps and institutional affiliations.

## Supplementary Material

Supplementary Information

## Figures and Tables

**Table 1:** Top 5 functional networks established from common top 50 PB-upregulated and top 50 PB-downregulated genes in Hep3B and HepG2 cells.

ID	Molecules in Network	Score	Focus Molecules	Associated Network Functions
1	alcohol group acceptor phosphotransferase, Ap1, ATF3, C/ebp, CCNE2, CDC7, Cdk, CDK1, CDKN2B, CENPF, Ck2, Cyclin A, Cyclin D, CYP2B6, DCK, E2f, EGR1, ETS, GOLM1, JUNB, MIR17HG, NCF2, NEK2, NR1H4, PEPCK, RAD51AP1, Rb, RNA polymerase II, Rxr, SERPINE1, SKA2, SKP2, TACC2, TIMP1, TTK	47	22	Cell Cycle, Cellular Growth and Proliferation, Cellular Development
2	AGER, ARHGEF2, ASB9, Beta Tubulin, CDKN2C, DKK1, DNMBP, EML1, FES, GABRR1, hexokinase, Histone h3, Histone h4, HNRNPA2B1, Hsp27, Hsp70, Hsp90, IGFBP1, MAP1B, MX1, NES, NPC1, p85 (pik3r), PACRG, PDE6G, PSIP1, RAB8B, SMC4, TOP2A, TUBA1A, TUBA3C/TUBA3D, TUBB3, TUBB2B, tubulin (complex), tubulin (family)	31	16	Cancer, Connective Tissue Disorders, Organismal Injury and Abnormalities
3	ABCG5, ACO1, ADRB2, AKR1C1/AKR1C2, AKR1D1, BHLHE40, Bhlhe41, BTG1, CIPC, CLIC4, CPB2, CYP2B6, DGAT2, ENDOG, FDPS, GLYCTK, HIF1A, HNF4A, IGFBP1, LPIN1, LPIN2, NAALAD2, NR3C1, NUDT6, RASD1, REL, RORC, SLC39A14, Slco1a1, STK19, TXNL4B, UGT2A3, ZDHHC9, ZHX1, ZHX3	28	15	Carbohydrate Metabolism, Lipid Metabolism, Small Molecule Biochemistry
4	ABCB4, ACSS1, ACSS2, ADAM8, ASPM, CCND1, CDC14B, CDK4/6, CLIC4, CTDSP1, DUSP5, ERK1/2, ESR1, FBXO4, FST, GPAM, IL17RB, KLF9, mir-30, mir-183, MIR17HG, MYC, Ncoa6, NDRG2, NECTIN3, PHLDB3, PPARA, PTGER3, PTPRH, SP2, SPAG4, STXBP1, TP53, TP53INP2, ZFP36L1	26	14	Cell Morphology, Tissue Morphology, Cancer
5	ACO1, ANP32E, ARGLU1, CENPE, CMTR1, COLGALT1, FAM169A, FDPS, FOS, GRHPR, HELLS, HSPA4L, HSPB8, LMNA, MAP1A, MBNL3, MIB1, MICAL2, NTRK1, NXF1, PATL1, PAXBP1, PCF11, PCNP, POGZ, RAPGEF1, RBM6, SCAF8, SEMA3A, SEMA3G, SMG1, SSUH2, SYNE1, TAOK1, TMEM57	24	13	Cancer, Organismal Injury and Abnormalities, Cellular Development
