# Supplementary material for: Aqueous extract of Polygonum bistorta modulates proteostasis by ROS-induced ER stress in human hepatoma cells
Source: Sci Rep. 2017 Jan 30;7:41437. doi: 10.1038/srep41437 (PMC5278379; doi:10.1038/srep41437)
Supplement: Supplementary Information [file srep41437-s1.pdf]

Aqueous extract of *Polygonum bistorta* modulates proteostasis by ROS-induced ER stress in human hepatoma cells

Yu-Huei Liu, Yui-Ping Weng, Hsuan-Yuan Lin, Sai-Wen Tang, Chao-Jung Chen, Chi-Jung Liang, Chung-Yu Ku and Jung-Yaw Lin*.

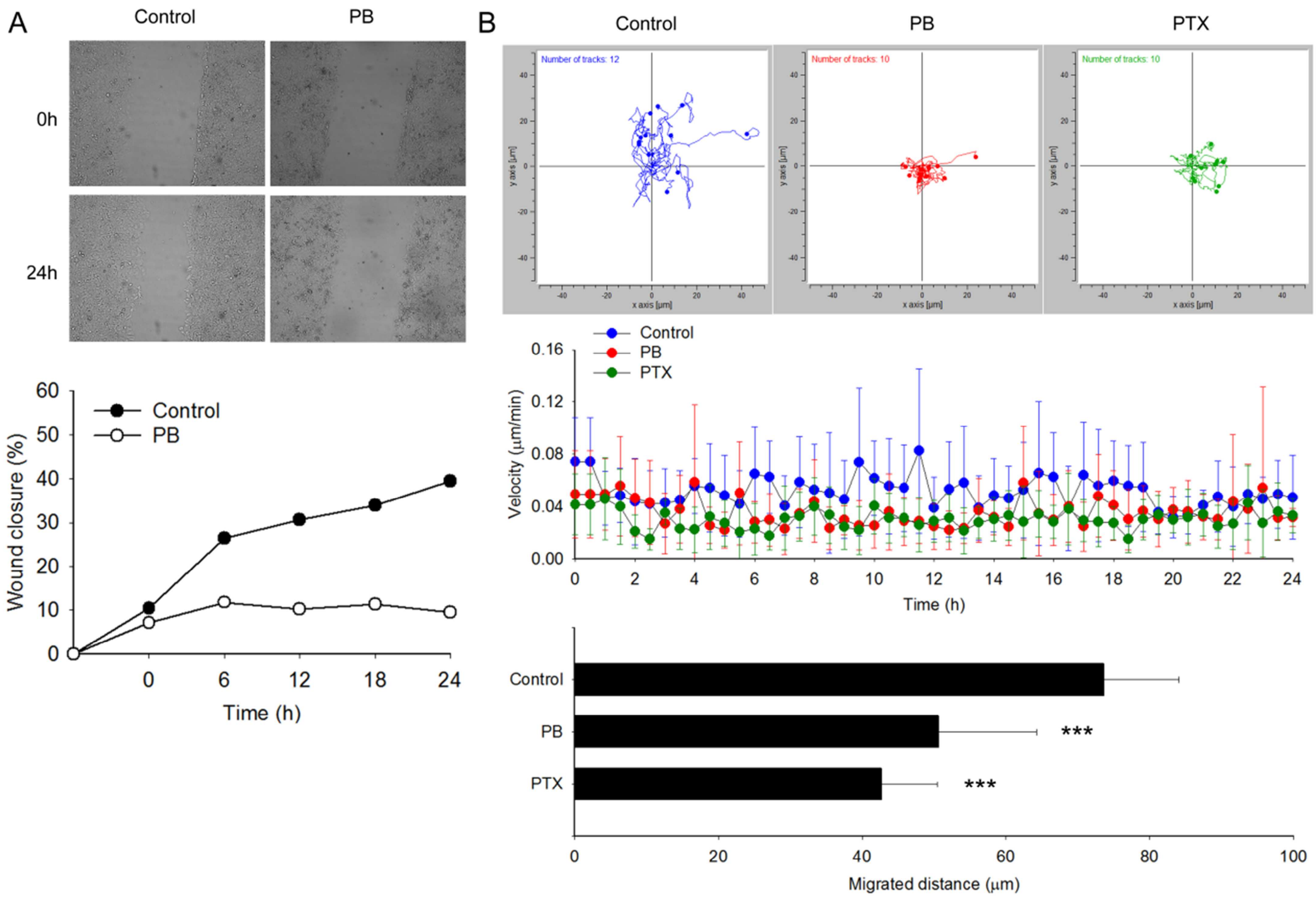

Supplementary Figure 1. PB inhibited cell migration. (A) Hep3B cells were treated for 24 h with 120 µg/mL PB. Images undergoing continuous morphological changes during wound healing were recorded. Percentage of wound closure was determined at 6 h intervals throughout the recording period. (B) Hep3B cells were treated for 24 h with 120 µg/mL PB or 2.0 µM PTX. Upper: individual trajectories were displayed with the initial point of each trajectory placed at the origin of the plot. Middle: average migration velocities were shown at 30 min intervals over 24 h. Lower: total migration distances were recorded. Data are means ± SD, with n ≥ 10. Data are presented from three independent experiments. ***$P < 0.001$, control versus PB- or PTX-treated cells.

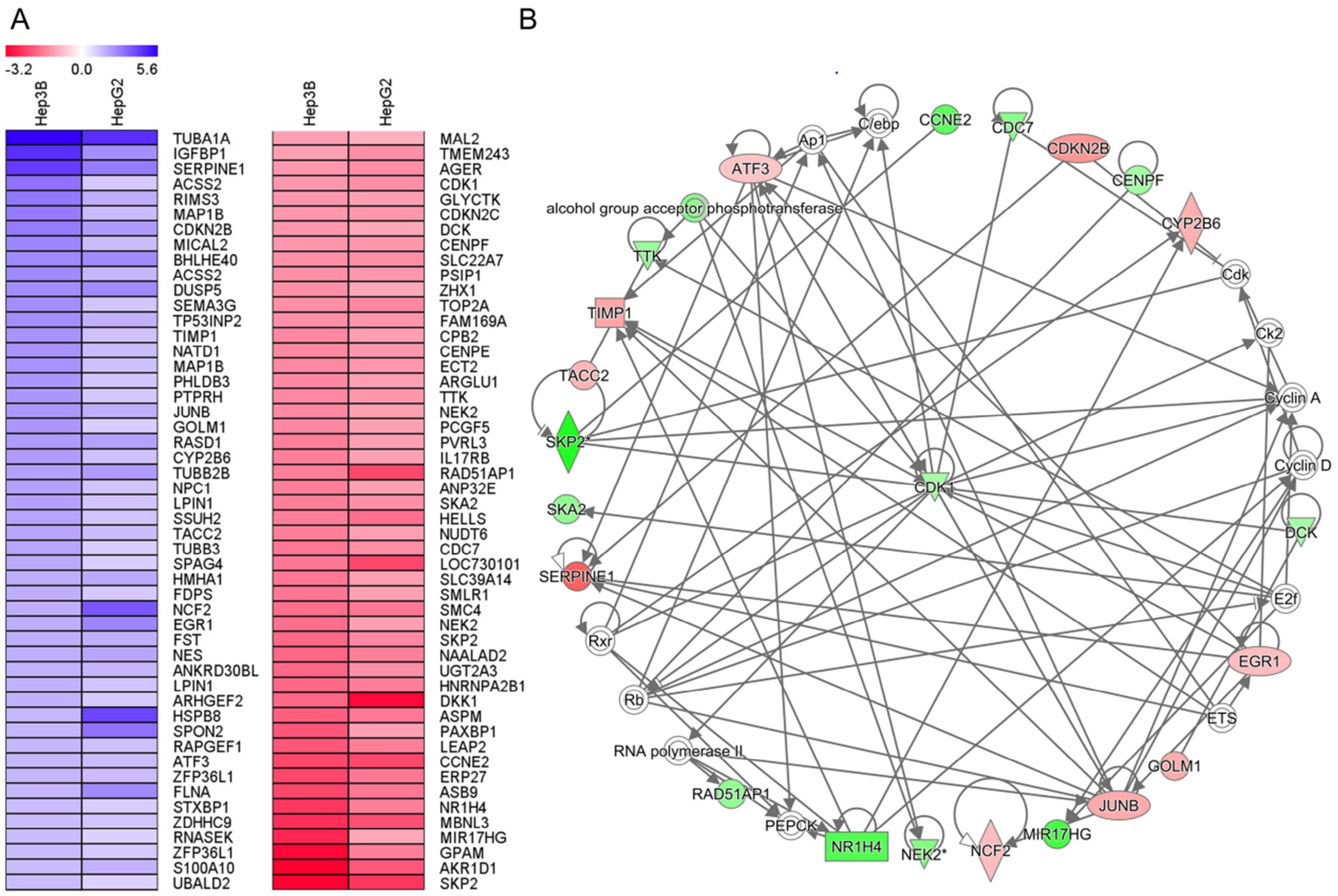

Supplementary Figure 2. Identification of PB transcriptional expression profile. (A) Heat map representing common PB-regulated genes (50 most upregulated, blue; 50 most downregulated, red) obtained from Hep3B and HepG2 cells treated with vehicle or PB (120 μg/mL, 6 h). (B) CDK1 and CDK1-target genes in the common top 50 PB up- and down-regulatory genes in Hep3B and HepG2 cells shown by Ingenuity pathway analysis. The colours of the nodes show the fold changes of the differentially expressed genes between PB-treated and control cells (red, upregulated genes; green, downregulated genes). Functional connections are indicated as arrows.

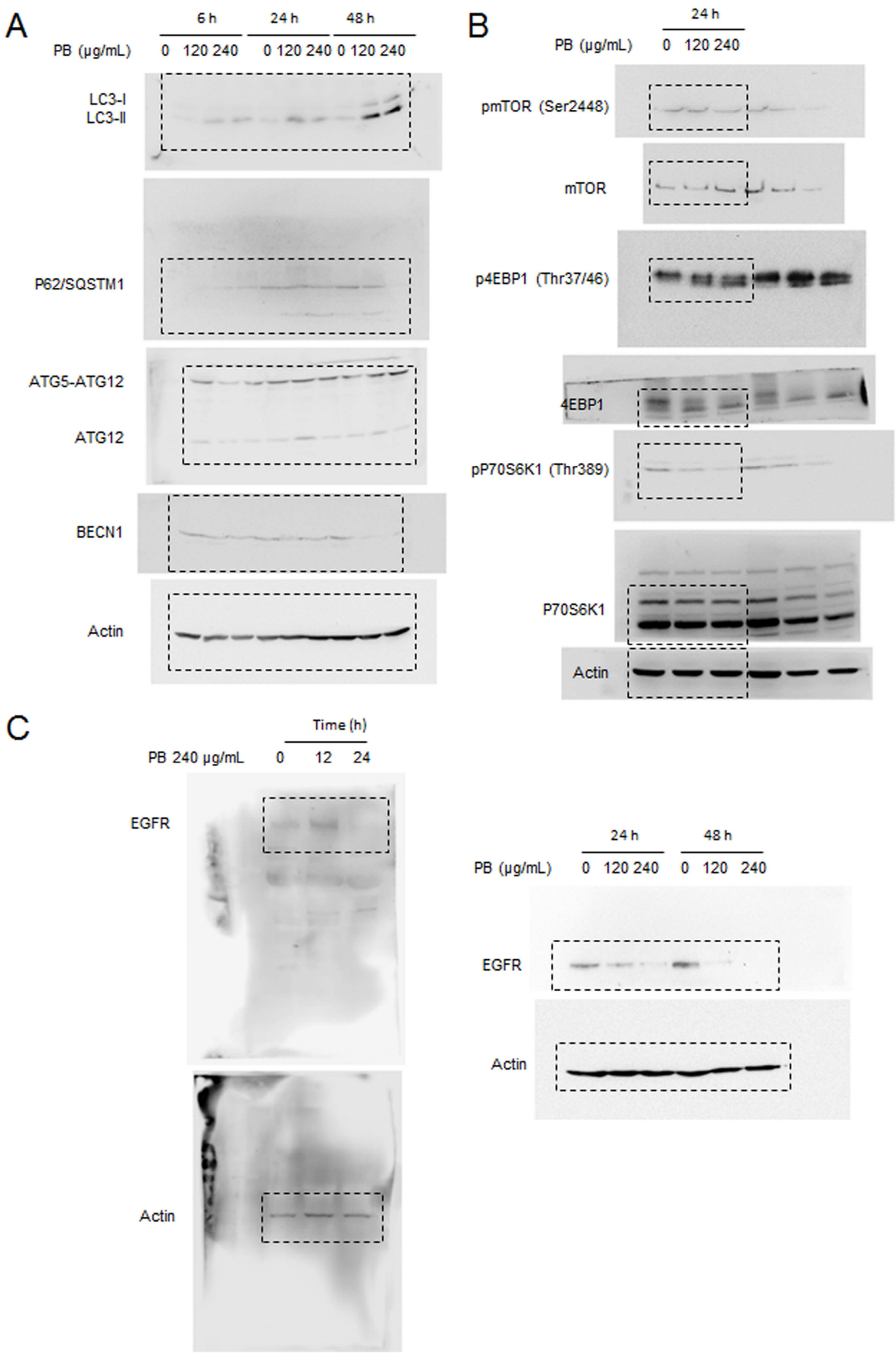

Supplementary Figure 3.

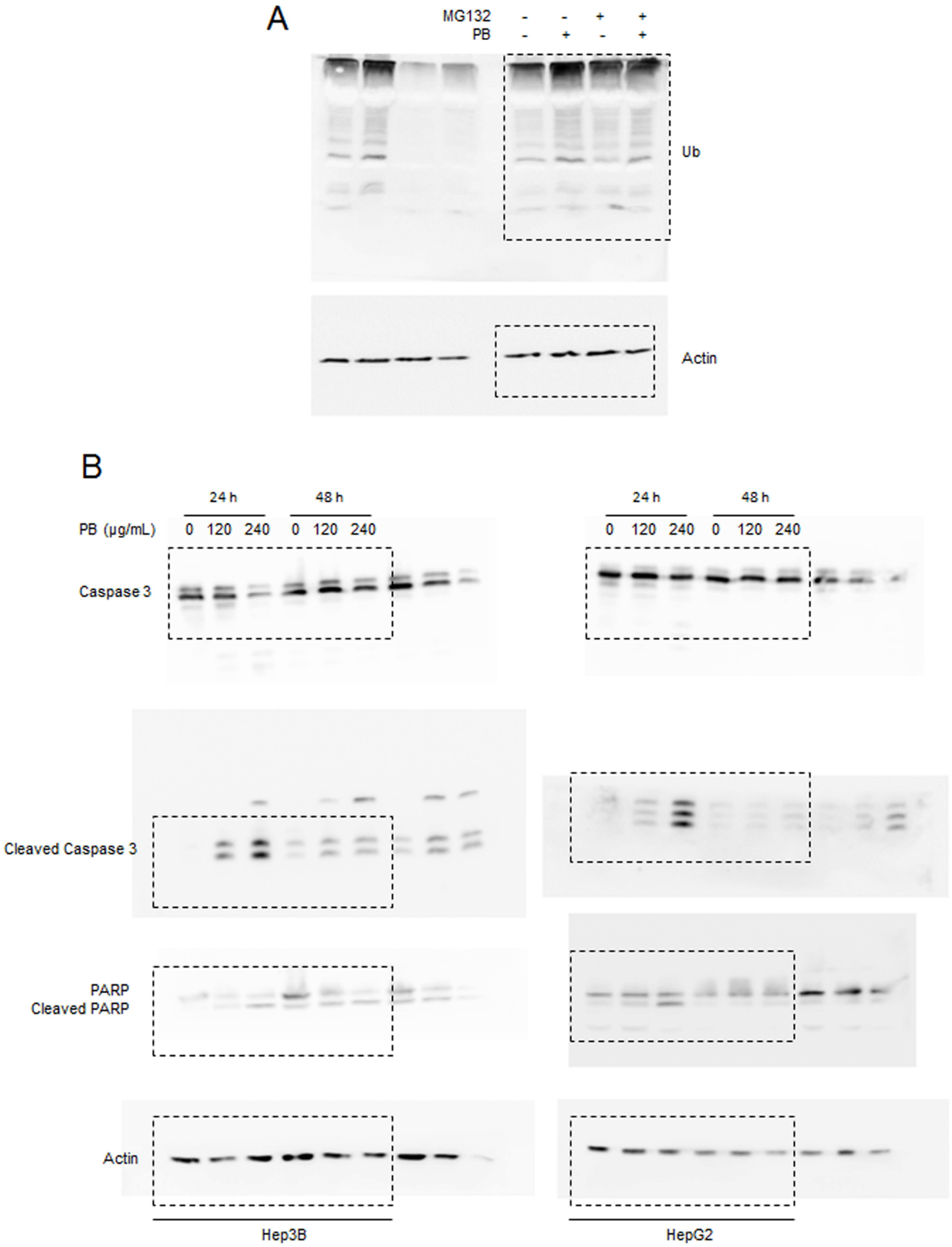

Supplementary Figure 4.

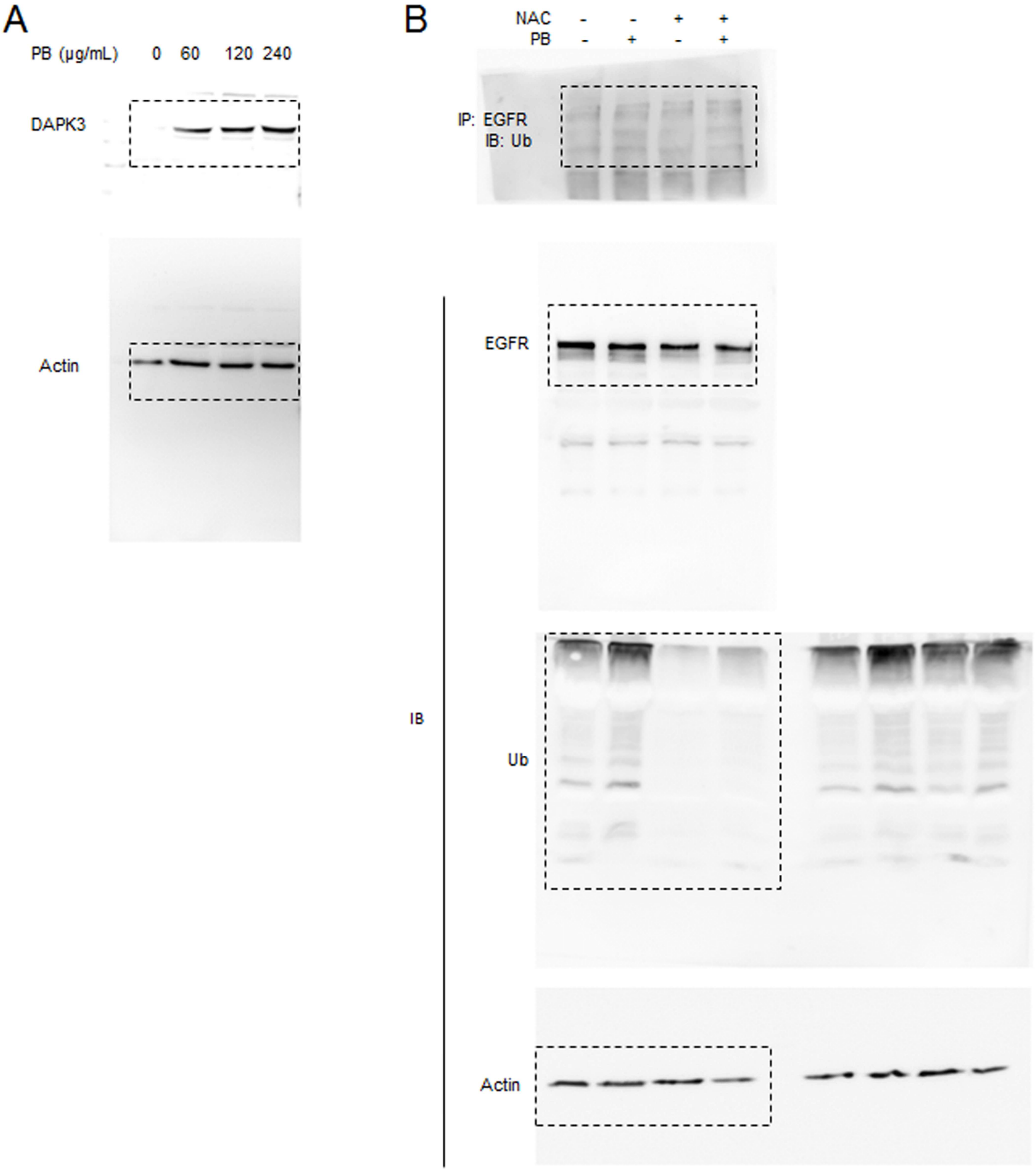

Supplementary Figure 5.

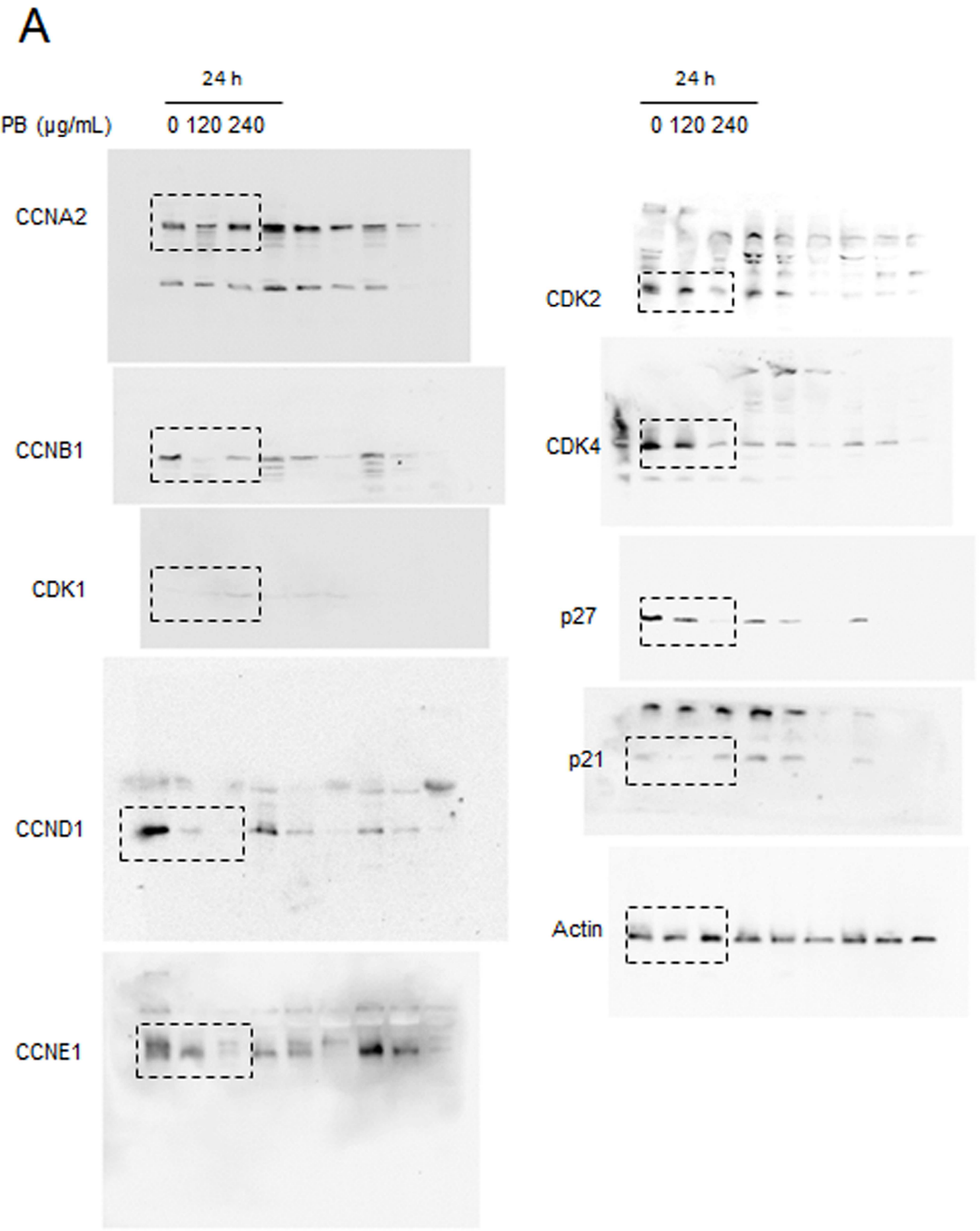

Supplementary Figure 6.

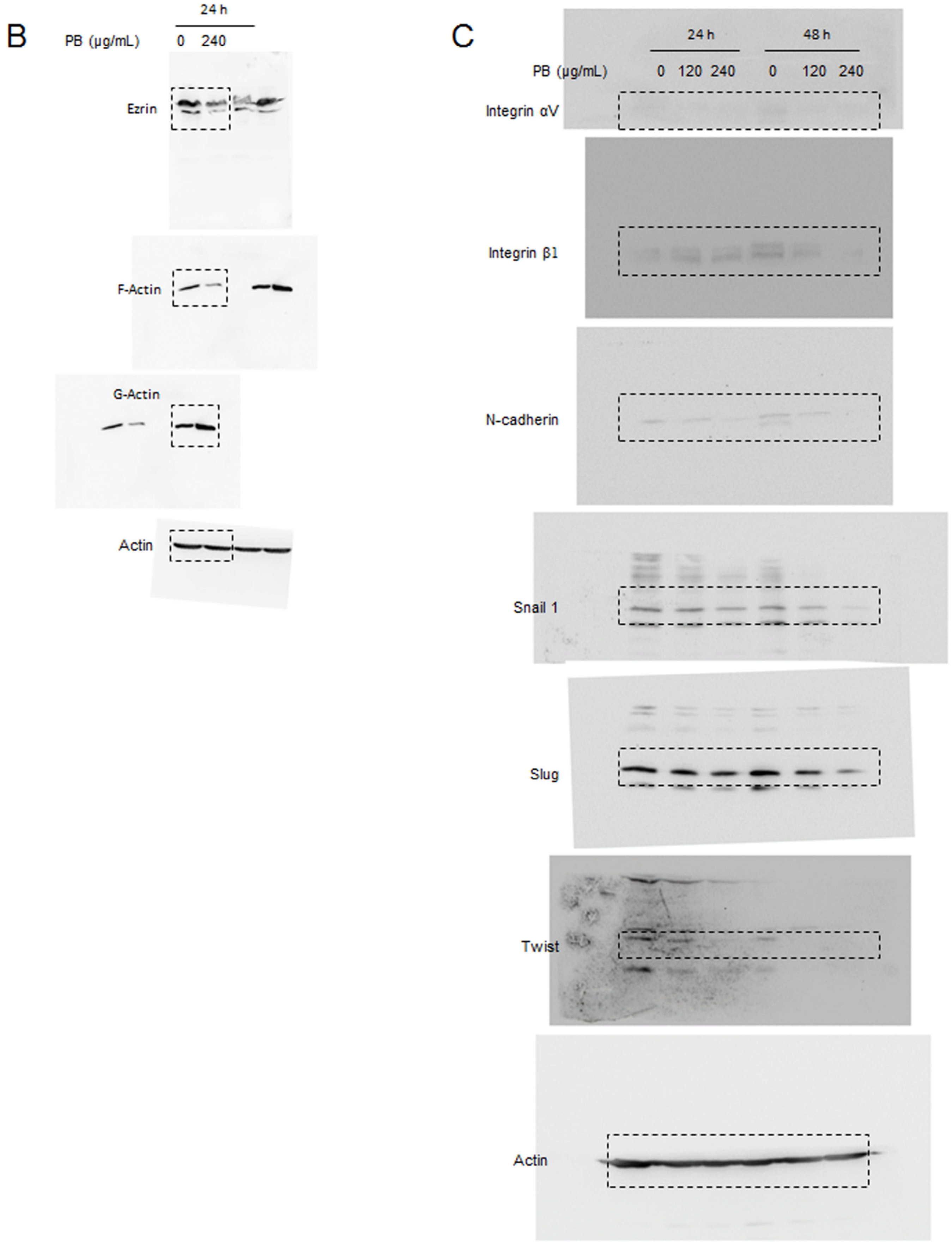

Supplementary Figure 6 (continued).

Supplementary Table S1. common top 50 *Polygonum bistorta* (PB)-upregulated and top 50 PB-downregulated genes in Hep3B and HepG2 cells.

				Exp Fold (Log 2 ratio)	
Symbol	Entrez Gene Name	Location	Type(s)	Hep3B	HepG2
TUBA1A	tubulin alpha 1a	Cytoplasm	other	5.6	4.6
IGFBP1	insulin like growth factor binding protein 1	Extracellular Space	other	4.6	2.5
SERPINE1	serpin family E member 1	Extracellular Space	other	4.3	2.9
ACSS2	acyl-CoA synthetase short-chain family member 2	Cytoplasm	enzyme	3.1	1.2
RIMS3	regulating synaptic membrane exocytosis 3	Plasma Membrane	other	3.0	1.8
MAP1B	microtubule associated protein 1B	Cytoplasm	other	2.9	1.5
CDKN2B	cyclin-dependent kinase inhibitor 2B	Nucleus	transcription regulator	2.9	2.2
MICAL2	microtubule associated monooxygenase, calponin and LIM domain containing 2	Cytoplasm	enzyme	2.7	1.5
BHLHE40	basic helix-loop-helix family member e40	Nucleus	transcription regulator	2.6	2.6
ACSS2	acyl-CoA synthetase short-chain family member 2	Cytoplasm	enzyme	2.6	1.6
DUSP5	dual specificity phosphatase 5	Nucleus	phosphatase	2.5	2.6
SEMA3G	semaphorin 3G	Cytoplasm	other	2.5	1.3
TP53INP2	tumor protein p53 inducible nuclear protein 2	Nucleus	other	2.5	1.7

TIMP1	TIMP metallopeptidase inhibitor 1	Extracellular Space	cytokine	2.4	1.4
NATD1	N-acetyltransferase domain containing 1	Other	other	2.4	1.5
MAP1B	microtubule associated protein 1B	Cytoplasm	other	2.4	1.4
PHLDB3	pleckstrin homology like domain family B member 3	Other	other	2.3	1.3
PTPRH	protein tyrosine phosphatase, receptor type H	Plasma Membrane	phosphatase	2.3	1.2
JUNB	jun B proto-oncogene	Nucleus	transcription regulator	2.3	1.8
GOLM1	golgi membrane protein 1	Cytoplasm	other	2.3	1.1
RASD1	ras related dexamethasone induced 1	Cytoplasm	enzyme	2.2	2.1
CYP2B6	cytochrome P450 family 2 subfamily B member 6	Cytoplasm	enzyme	2.2	1.4
TUBB2B	tubulin beta 2B class IIb	Cytoplasm	other	2.1	2.2
NPC1	Niemann-Pick disease, type C1	Cytoplasm	transporter	2.1	1.3
LPIN1	lipin 1	Nucleus	phosphatase	2.1	1.3
SSUH2	ssu-2 homolog (C. elegans)	Cytoplasm	other	2.0	1.3
TACC2	transforming acidic coiled-coil containing protein 2	Nucleus	other	2.0	1.5
TUBB3	tubulin beta 3 class III	Cytoplasm	other	2.0	1.1
SPAG4	sperm associated antigen 4	Cytoplasm	other	1.9	1.1
HMHA1	histocompatibility (minor) HA-1	Cytoplasm	transporter	1.8	1.9
FDPS	farnesyl diphosphate synthase	Cytoplasm	enzyme	1.8	1.2
NCF2	neutrophil cytosolic factor 2	Cytoplasm	enzyme	1.8	3.7
EGR1	early growth response 1	Nucleus	transcription regulator	1.8	2.7

FST	follistatin	Extracellular Space	other	1.8	1.8
NES	nestin	Other	other	1.8	2.0
ANKRD30BL	ankyrin repeat domain 30B-like	Other	other	1.7	1.6
LPIN1	lipin 1	Nucleus	phosphatase	1.7	1.3
ARHGEF2	Rho/Rac guanine nucleotide exchange factor 2	Cytoplasm	other	1.7	1.2
HSPB8	heat shock protein family B (small) member 8	Cytoplasm	kinase	1.6	4.1
SPON2	spondin 2	Extracellular Space	other	1.6	3.1
RAPGEF1	Rap guanine nucleotide exchange factor 1	Cytoplasm	other	1.6	1.4
ATF3	activating transcription factor 3	Nucleus	transcription regulator	1.6	1.5
ZFP36L1	ZFP36 ring finger protein-like 1	Nucleus	transcription regulator	1.6	1.5
FLNA	filamin A	Cytoplasm	other	1.6	2.6
STXBP1	syntaxin binding protein 1	Cytoplasm	transporter	1.5	1.1
ZDHHC9	zinc finger DHHC-type containing 9	Cytoplasm	enzyme	1.5	1.3
RNASEK	ribonuclease K	Other	peptidase	1.5	1.0
ZFP36L1	ZFP36 ring finger protein-like 1	Nucleus	transcription regulator	1.5	1.2
S100A10	S100 calcium binding protein A10	Cytoplasm	other	1.5	1.7
UBALD2	UBA like domain containing 2	Other	other	1.5	1.0
MAL2	mal, T-cell differentiation protein 2 (gene/pseudogene)	Plasma Membrane	transporter	-1.2	-1.0

TMEM243	transmembrane protein 243	Other	other	-1.2	-1.4
AGER	advanced glycosylation end product-specific receptor	Plasma Membrane	transmembrane receptor	-1.3	-1.4
CDK1	cyclin-dependent kinase 1	Nucleus	kinase	-1.3	-1.4
GLYCTK	glycerate kinase	Cytoplasm	kinase	-1.3	-1.2
CDKN2C	cyclin-dependent kinase inhibitor 2C	Nucleus	transcription regulator	-1.3	-1.3
DCK	deoxycytidine kinase	Nucleus	kinase	-1.3	-1.1
CENPF	centromere protein F	Nucleus	other	-1.3	-1.3
PSIP1	PC4 and SFRS1 interacting protein 1	Nucleus	other	-1.4	-1.4
SLC22A7	solute carrier family 22 member 7	Plasma Membrane	transporter	-1.4	-1.3
ZHX1	zinc fingers and homeoboxes 1	Nucleus	transcription regulator	-1.4	-1.1
TOP2A	topoisomerase (DNA) II alpha	Nucleus	enzyme	-1.4	-1.5
FAM169A	family with sequence similarity 169 member A	Other	other	-1.4	-1.3
CPB2	carboxypeptidase B2	Extracellular Space	peptidase	-1.5	-1.2
CENPE	centromere protein E	Nucleus	other	-1.5	-1.3
ECT2	epithelial cell transforming 2	Cytoplasm	other	-1.5	-1.3
ARGLU1	arginine and glutamate rich 1	Other	other	-1.5	-1.2
TTK	TTK protein kinase	Nucleus	kinase	-1.5	-1.3
NEK2	NIMA related kinase 2	Cytoplasm	kinase	-1.5	-1.2
PCGF5	polycomb group ring finger 5	Cytoplasm	other	-1.5	-1.2

NECTIN3	nectin cell adhesion molecule 3	Plasma Membrane	other	-1.6	-1.2
IL17RB	interleukin 17 receptor B	Plasma Membrane	transmembrane receptor	-1.6	-1.2
RAD51AP1	RAD51 associated protein 1	Nucleus	other	-1.6	-2.3
ANP32E	acidic nuclear phosphoprotein 32 family member E	Nucleus	other	-1.6	-1.2
SKA2	spindle and kinetochore associated complex subunit 2	Nucleus	other	-1.6	-1.4
HELLS	helicase, lymphoid-specific	Nucleus	enzyme	-1.6	-1.8
NUDT6	nudix hydrolase 6	Extracellular Space	growth factor	-1.6	-1.2
CDC7	cell division cycle 7	Nucleus	kinase	-1.7	-1.4
LOC730101	uncharacterized LOC730101	Other	other	-1.7	-2.3
SLC39A14	solute carrier family 39 member 14	Plasma Membrane	transporter	-1.7	-1.2
SMLR1	small leucine-rich protein 1	Other	other	-1.7	-1.2
SMC4	structural maintenance of chromosomes 4	Nucleus	transporter	-1.8	-1.7
NEK2	NIMA related kinase 2	Cytoplasm	kinase	-1.8	-1.2
SKP2	S-phase kinase-associated protein 2, E3 ubiquitin protein ligase	Nucleus	enzyme	-1.9	-1.5
NAALAD2	N-acetylated alpha-linked acidic dipeptidase 2	Plasma Membrane	peptidase	-1.9	-1.6

UGT2A3	UDP glucuronosyltransferase family 2 member A3	Other	enzyme	-1.9	-1.4
HNRNPA2B1	heterogeneous nuclear ribonucleoprotein A2/B1	Nucleus	other	-1.9	-1.6
DKK1	dickkopf WNT signaling pathway inhibitor 1	Extracellular Space	growth factor	-1.9	-3.1
ASPM	abnormal spindle microtubule assembly	Nucleus	other	-2.0	-1.7
PAXBP1	PAX3 and PAX7 binding protein 1	Nucleus	other	-2.1	-1.2
LEAP2	liver expressed antimicrobial peptide 2	Extracellular Space	other	-2.1	-1.6
CCNE2	cyclin E2	Nucleus	other	-2.3	-2.3
ERP27	endoplasmic reticulum protein 27	Other	other	-2.3	-1.7
ASB9	ankyrin repeat and SOCS box containing 9	Nucleus	transcription regulator	-2.3	-1.7
NR1H4	nuclear receptor subfamily 1 group H member 4	Nucleus	ligand-dependent nuclear receptor	-2.5	-1.6
MBNL3	muscleblind like splicing regulator 3	Nucleus	other	-2.6	-2.2
MIR17HG	miR-17-92a-1 cluster host gene	Other	other	-2.7	-1.1
GPAM	glycerol-3-phosphate acyltransferase, mitochondrial	Cytoplasm	enzyme	-3.1	-1.6
AKR1D1	aldo-keto reductase family 1, member D1	Cytoplasm	enzyme	-3.2	-2.1
SKP2	S-phase kinase-associated protein 2, E3 ubiquitin protein ligase	Nucleus	enzyme	-3.2	-2.5

Supplementary Table S2. Target molecules and drugs for top 50 *Polygonum bistorta* (PB)-upregulated and top 50 PB-downregulated genes in Hep3B and HepG2 cells.

Entrez Gene ID	Symbol	Exp Fold Change (Log 2 ratio)	Location	Type(s)	Biomarker Application(s)	Drug(s)
3484	IGFBP1	4.6	Extracellular Space	other	diagnosis, efficacy	
5054	SERPINE1	4.3	Extracellular Space	other	diagnosis, disease progression, efficacy, prognosis, safety	drotrecogin alfa
1030	CDKN2B	2.9	Nucleus	transcription regulator	diagnosis, prognosis	
7076	TIMP1	2.4	Extracellular Space	cytokine	diagnosis, disease progression, efficacy, prognosis	
3726	JUNB	2.3	Nucleus	transcription regulator	response to therapy	
51280	GOLM1	2.3	Cytoplasm	other	Diagnosis	
1555	CYP2B6	2.2	Cytoplasm	enzyme	efficacy, prognosis	
10579	TACC2	2.0	Nucleus	other	Diagnosis	

10381	TUBB3	2.0	Cytoplasm	other	response to therapy	epothilone B, ixabepilone, colchicine/probenecid, larotaxel, ABT-751, eribulin, simotaxel, davunetide, vintafolide, milataxel, cevipabulin, gemcitabine/paclitaxel, docetaxel/prednisone, capecitabine/docetaxel, paclitaxel/trastuzumab, capecitabine/ixabepilone, cyclophosphamide/prednisone/vincristine, docetaxel/hydrocortisone, cyclophosphamide/docetaxel, gemcitabine/vinorelbine, cyclophosphamide/daunorubicin/imatinib/prednisone/vincristine, cyclophosphamide/topotecan/vincristine, docetaxel/gemcitabine, docetaxel/gemcitabine/vincristine, irinotecan/vincristine, irinotecan/temozolomide/vincristine, bevacizumab/paclitaxel, cyclophosphamide/docetaxel/epirubicin/5-fluorouracil/trastuzumab, docetaxel/trastuzumab, trastuzumab/vinorelbine, gemcitabine/oxaliplatin/paclitaxel, cyclophosphamide/epirubicin/vincristine,

						docetaxel/irinotecan, docetaxel/5-fluorouracil/oxaliplatin, capecitabine/docetaxel/gemcitabine, L-asparaginase/prednisone/vincristine, cyclophosphamide/etoposide/prednisone/rituximab/ vincristine, cyclophosphamide/vinorelbine, cyclophosphamide/mitoxantrone/prednisone/vincristine, cyclophosphamide/etoposide/prednisone/vincristine, cyclophosphamide/prednisone/rituximab/vincristine, cyclophosphamide/mitoxantrone/prednisone/rituximab/vincristine, plinabulin, docetaxel/epirubicin, docetaxel/paclitaxel, epirubicin/paclitaxel, bevacizumab/paclitaxel/topotecan, paclitaxel/topotecan, bevacizumab/docetaxel, cyclophosphamide/prednisolone/vincristine, cyclophosphamide/prednisolone/rituximab/vincristine, cyclophosphamide/epirubicin/5-fluorouracil/vinorelbine, cyclophosphamide/epirubicin/5-fluorouracil/paclitaxel/trastuzumab, cyclophosphamide/epirubicin/5-fluorouracil/paclitax

						el, cyclophosphamide/docetaxel/epirubicin/5-fluorouracil, cyclophosphamide/docetaxel/trastuzumab, cyclophosphamide/gemcitabine/prednisolone/rituximab/vincristine, cyclophosphamide/epirubicin/prednisone/vincristine, BMS-275183, docetaxel, vinflunine, vinorelbine, vincristine, vinblastine, paclitaxel, podophyllotoxin, colchicine
1958	EGR1	1.8	Nucleus	transcription regulator	Diagnosis	
10417	SPON2	1.6	Extracellular Space	other	Diagnosis	
177	AGER	-1.3	Plasma Membrane	transmembrane receptor	diagnosis, efficacy	2-O,3-O-desulfated heparin
1633	DCK	-1.3	Nucleus	kinase	efficacy, response to therapy	cytarabine/fludarabine phosphate, cladribine/cytarabine/daunorubicin, cladribine/cytarabine/filgrastim, cytarabine/filgrastim/fludarabine phosphate, cladribine/cytarabine/filgrastim/mitoxantrone, fludarabine phosphate/rituximab, cladribine/rituximab,

						cytarabine/filgrastim/fludarabine phosphate/idarubicin, dexamethasone/fludarabine phosphate/mitoxantrone, alemtuzumab/fludarabine phosphate, fludarabine phosphate/mitoxantrone/rituximab, dexamethasone/fludarabine phosphate/mitoxantrone/rituximab, fludarabine phosphate/mitoxantrone, chlorambucil/cladribine, cladribine/cyclophosphamide, fludarabine phosphate, cladribine
11168	PSIP1	-1.4	Nucleus	other	disease progression	
7153	TOP2A	-1.4	Nucleus	enzyme	diagnosis, efficacy, prognosis, response to therapy	novobiocin, etoposide, teniposide, CPI-0004Na, becatecarin, elsamitrucin, AQ4N, elomotecan, tafluposide, fleroxacin, cyclophosphamide/epirubicin/5-fluorouracil, cytarabine/daunorubicin, finafloxacin, hydrocortisone/mitoxantrone, mitoxantrone/prednisone, cladribine/cytarabine/daunorubicin, cytarabine/daunorubicin/tretinoin, cytarabine/idarubicin, idarubicin/mitoxantrone/tretinoin, arsenic

						trioxide/daunorubicin/tretinoin, cytarabine/etoposide, cytarabine/etoposide/mitoxantrone, cytarabine/mitoxantrone, daunorubicin/tretinoin, idarubicin/tretinoin, bortezomib/dexamethasone/doxorubicin, cisplatin/doxorubicin/methotrexate, cyclophosphamide/epirubicin, cyclophosphamide/etoposide, cisplatin/doxorubicin/ifosfamide/methotrexate, cisplatin/doxorubicin, cyclophosphamide/docetaxel/epirubicin/5-fluorouracil/trastuzumab, clofarabine/cyclophosphamide/etoposide, cyclophosphamide/epirubicin/vincristine, bortezomib/doxorubicin, carboplatin/doxorubicin, cladribine/cytarabine/filgrastim/mitoxantrone, epirubicin/5-fluorouracil/oxaliplatin, dacarbazine/doxorubicin, cytarabine/filgrastim/fludarabine phosphate/idarubicin, capecitabine/epirubicin/oxaliplatin, doxorubicin/ifosfamide,

						cladribine/cytarabine/filgrastim/idarubicin, doxorubicin/streptozocin, dacarbazine/doxorubicin/ifosfamide, dexamethasone/fludarabine phosphate/mitoxantrone, cyclophosphamide/etoposide/prednisone/rituximab/vincristine, cyclophosphamide/fludarabine phosphate/mitoxantrone/rituximab, fludarabine phosphate/mitoxantrone/rituximab, cyclophosphamide/mitoxantrone/prednisone/vincristine, cyclophosphamide/etoposide/prednisone/vincristine, dexamethasone/fludarabine phosphate/mitoxantrone/rituximab, chlorambucil/mitoxantrone/prednisone, fludarabine phosphate/mitoxantrone, cyclophosphamide/fludarabine phosphate/mitoxantrone, alemtuzumab/cyclophosphamide/fludarabine phosphate/mitoxantrone, cyclophosphamide/mitoxantrone/prednisone/rituximab/vincristine, cladribine/cytarabine/filgrastim/idarubicin/plerixafor

						, doxorubicin/tretinoin, cytarabine/doxorubicin, chlorambucil/mitoxantrone/prednisone/rituximab, docetaxel/epirubicin, doxorubicin/paclitaxel, epirubicin/paclitaxel, arsenic trioxide/idarubicin/tretinoin, clofarabine/filgrastim/idarubicin, clofarabine/cytarabine/filgrastim/idarubicin, cyclophosphamide/epirubicin/5-fluorouracil/vinorelbine, cyclophosphamide/epirubicin/5-fluorouracil/paclitaxel/trastuzumab, cytarabine/idarubicin/sorafenib, cyclophosphamide/epirubicin/5-fluorouracil/paclitaxel, cyclophosphamide/docetaxel/epirubicin/5-fluorouracil, cyclophosphamide/epirubicin/prednisone/vincristine, mitoxantrone, amsacrine, norfloxacin, dexrazoxane, tirapazamine, TAS-103, gatifloxacin, valrubicin, gemifloxacin, nemorubicin, nalidixic acid, levofloxacin, epirubicin, doxorubicin, idarubicin, daunorubicin, podophyllotoxin, cis-sparfloxacin

22943	DKK1	-1.9	Extracellular Space	growth factor	efficacy, prognosis	
9971	NR1H4	-2.5	Nucleus	ligand-dependent nuclear receptor	diagnosis	
6502	SKP2	-3.2	Nucleus	enzyme	diagnosis	

Methods

Time-lapse microscopy

To assess the effects of PB on wound healing, 3×10^4 cells were placed on each side of a Culture-Insert 2 Well (Ibidi, Munich, Germany) with a 500-μm separation for 24 h, and replaced in serum-free medium for a further 16 h. Cells were incubated in DMEM with 10% FBS plus PB (240 μg/mL) or water control for 24 h after removal of the insert. For migration velocities, quantities of 5×10^5 Hep3B cells were seeded onto 3.5 cm dishes for 24 h and replaced in serum-free medium for a further 24 h. Cells were incubated in DMEM with 10% FBS plus PB (240 μg/mL), PTX (2.0 μM), or water control at the start of 24 h of time-lapse microscopy. A live cell movie analyzer (NanoEntek) was used to capture images every 30 min. An average of at least 10 subsequent cell centroid displacements per 30 min from two consecutive images was analyzed to determine cell velocities of migration by using the Manual Tracking plugin for ImageJ software (National Institutes of Health, USA).

RNA extraction, microarray analysis, and network analysis

Total RNA from Hep3B or HepG2 cells treated with PB or water control for 24 h was extracted using Trizol reagent (Invitrogen) and an RNeasy Mini kit (Qiagen, Venlo, Netherlands) according to the manufacturer's instructions. RNA quantity and purity were assessed by NanoDrop ND-1000 followed by Agilent RNA 6000 Nano assay. A lack of genomic DNA contamination was confirmed by gel electrophoresis. Total RNA with absorbance ratios A260/A280 ≥ 1.8 and A260/A230 ≥ 1.5, as well as RIN value > 6, indicated RNA of acceptable integrity. Target preparation coupled with NHS-Cy5 (GE Amersham, USA) was generated as amino-allyl antisense RNA (aRNA) by using an Amino Allyl MessageAmp II aRNA Amplification Kit (Ambion, USA). Purified, labeled aRNA was quantified using a NanoDrop ND-1000 with Cy5 incorporation efficiency > 15 dye molecules/1000 nt. Quantities of 5 μg of each labeled aRNA were used for hybridization at 50°C for 16 h to the whole genome

Human OneArray® (version HOA 7.1, Phalanx Biotech Group, Taiwan) containing 28,264 well-characterized genes. The arrays were scanned using an Axon 4000B scanner (Molecular Devices, USA) followed by analyzed with Genepix software (Molecular Devices USA). The interaction networks as well as molecule and drug analysis were generated by Ingenuity pathway analysis (IPA) software version 01-07.
